# Embryonic osteocalcin signaling determines lifelong adrenal steroidogenesis and homeostasis in the mouse

**DOI:** 10.1172/JCI153752

**Published:** 2022-02-15

**Authors:** Vijay K. Yadav, Julian M. Berger, Parminder Singh, Perumal Nagarajan, Gerard Karsenty

**Affiliations:** 1 Department of Genetics and Development, Columbia University, New York, New York, USA.; 2 National Institute of Immunology, New Delhi, India.

**Keywords:** Bone Biology, Metabolism, Mouse models

## Abstract

Through their ability to regulate gene expression in most organs, glucocorticoid (GC) hormones influence numerous physiological processes and are therefore key regulators of organismal homeostasis. In bone, GC hormones inhibit expression of the hormone *Osteocalcin* for poorly understood reasons. Here, we show that in a classical endocrine feedback loop, osteocalcin in return enhanced the biosynthesis of GC as well as mineralocorticoid hormones (adrenal steroidogenesis) in rodents and primates. Conversely, inactivation of osteocalcin signaling in adrenal glands significantly impaired adrenal growth and steroidogenesis in mice. Embryo-made osteocalcin was necessary for normal *Sf1* expression in fetal adrenal cells and adrenal cell steroidogenic differentiation and therefore determined the number of steroidogenic cells present in the adrenal glands of adult animals. Embryonic, not postnatal, osteocalcin also governed adrenal growth, adrenal steroidogenesis, blood pressure, electrolyte equilibrium, and the rise in circulating corticosterone levels during the acute stress response in adult offspring. This osteocalcin-dependent regulation of adrenal development and steroidogenesis occurred even in the absence of a functional hypothalamus/pituitary/adrenal axis and explains why osteocalcin administration during pregnancy promoted adrenal growth and steroidogenesis and improved the survival of adrenocorticotropic hormone signaling–deficient animals. This study reveals that a bone-derived embryonic hormone influences lifelong adrenal functions and organismal homeostasis in the mouse.

## Introduction

The adrenal gland, an organ of fundamental importance in mammals, is composed of 2 distinct areas. The inner medulla is a neural crest derivative that produces the catecholamines norepinephrine and epinephrine (1). The outer cortex is of mesodermal origin and is arranged in 3 distinct zones, each synthesizing a different steroid hormone (1–4). The zona glomerulosa (zG) synthesizes the mineralocorticoid hormone aldosterone; the zona fasciculata (zF) synthesizes glucocorticoid (GC) hormones, corticosterone in rodents, and cortisol in primates; and the zona reticularis (zR), which is absent in rodents, synthesizes sulfate of dehydroepiandrostreonediones (DHEAs) (1–3).

During embryogenesis, adrenal development is initiated and largely controlled by the transcription factor steroidogenic factor 1 (SF1) (1, 5). *Sf1-*expressing fetal adrenal cells give rise to *Gli1*-positive, *Sf1*-negative nonsteroidogenic adrenocortical progenitor cells located in the capsule surrounding the developing gland (1, 5). These adrenocortical progenitor cells differentiate into *Sf1*-positive aldosterone-producing cells that move centripetally. GC-producing cells originate largely from aldosterone-producing ones through lineage conversion, a process that is also dependent on SF1 (1, 5).

Aldosterone and GC differ not only in their cells of origin but also in their regulation of synthesis and functions. Aldosterone biosynthesis is mainly regulated by extracellular potassium (K^+^) concentration and the renin angiotensin system, in which the kidney-derived protease renin triggers multiple protein conversion events, which culminate in the generation of angiotensin 2. After binding to its receptors on cells of the zG, angiotensin 2 favors aldosterone biosynthesis (6). GC biosynthesis is controlled by hypothalamic-pituitary inputs in what defines the hypothalamic/pituitary/adrenal (HPA) axis. Hypothalamus-derived corticotrophin-releasing hormone (CRH) promotes the synthesis of adrenocorticotropic hormone (ACTH) in the pituitary gland. ACTH signals in cells of the zF through the melanocortin 2 receptor (Mc2r) to favor GC synthesis and adrenal growth (7). Adrenal development occurs in part independently of pituitary influences, which suggests that other yet-to-be-identified hormones may regulate this process (8, 9).

Aldosterone regulates extracellular fluid and electrolyte homeostasis and therefore influences blood pressure (6, 10). As for GCs, they affect numerous physiological processes through their ability to modulate gene expression in a wide array of cell types (11, 12). In the skeleton, GCs are powerful inhibitors of *Osteocalcin* expression in osteoblasts, a regulation that was puzzling until evidence accumulated that osteocalcin is a hormone (13, 14). Indeed, given the endocrine identity of osteocalcin, the inhibition of its expression by GC suggests that, through a classical feedback loop, osteocalcin may be a previously unappreciated physiological regulator of GC biosynthesis. That osteocalcin regulates the biosynthesis of several other hormones adds further credence to this hypothesis (13).

Here, we show that osteocalcin is necessary (in mice) and sufficient (in mice and monkeys) for the biosynthesis of both GC and aldosterone (adrenal steroidogenesis). During embryogenesis, osteocalcin signals in the developing adrenal glands to promote *Sf1* expression, the entire differentiation program of fetal adrenal cells into nonsteroidogenic adrenocortical progenitor cells, and the differentiation of these latter cells into steroidogenic ones. As a result, embryo-derived osteocalcin determines the number of steroidogenic cells, adrenal growth, steroidogenesis, and several parameters of organismal homeostasis in adult offspring. None of these events or parameters of homeostasis are influenced by postnatal osteocalcin. This osteocalcin regulation of adrenal steroidogenesis occurs independently of the HPA axis and can be harnessed to induce adrenal growth and steroidogenesis and prevent perinatal death in embryos and mice lacking ACTH signaling. Hence, an embryo-derived hormone regulates adrenal steroidogenesis and organismal homeostasis postnatally.

## Results

### Osteocalcin is sufficient to increase adrenal steroidogenesis in rodents and primates.

**Figure 1:**
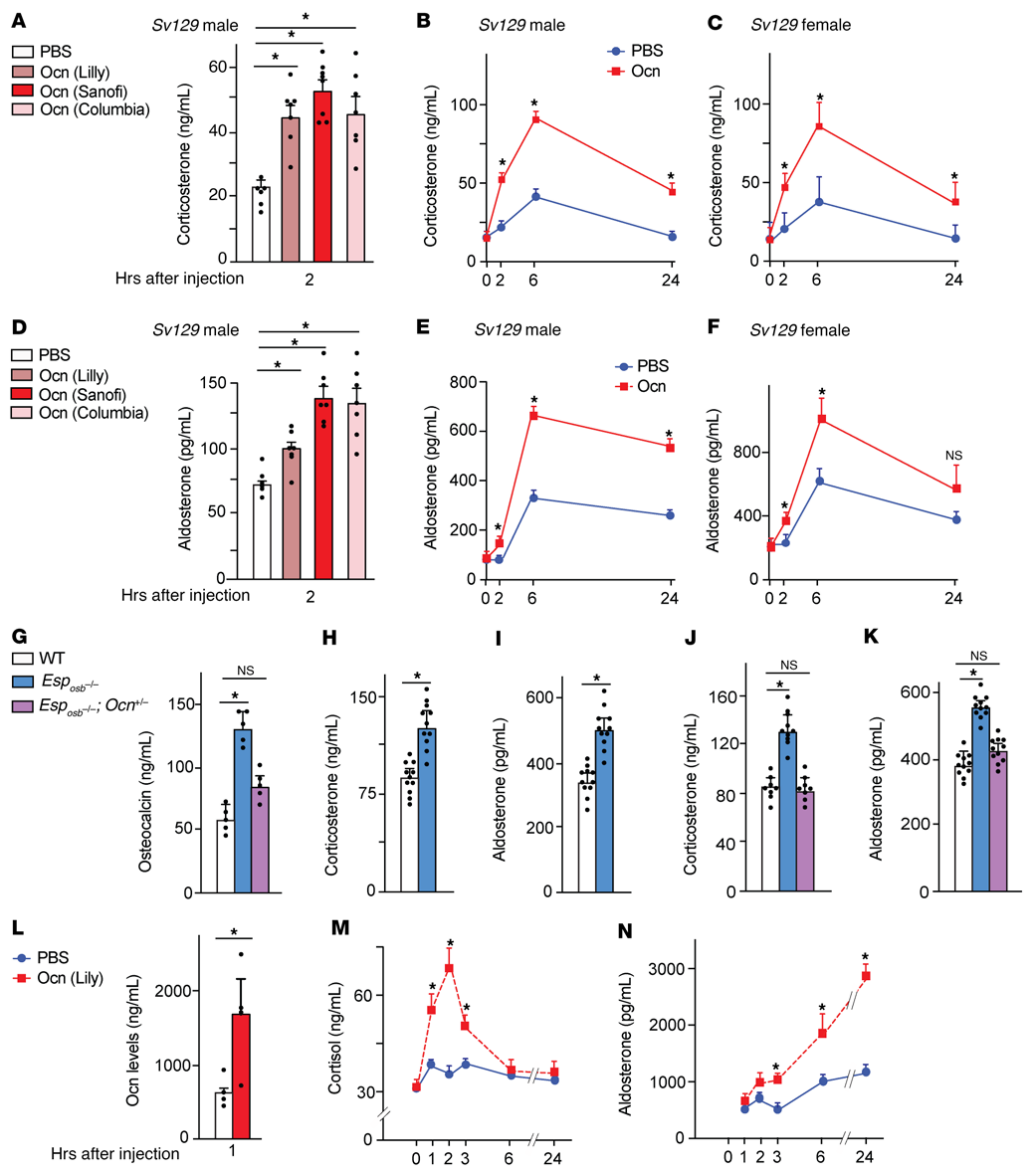
Osteocalcin increases circulating GCs and aldosterone in mice and monkeys. (**A**) Circulating corticosterone levels 2 hours after injection of vehicle or recombinant osteocalcin (Ocn) from different sources (30 ng/g body weight) at 1200 hours in 2-month-old *Sv129* male WT mice. (**B** and **C**) Circulating corticosterone levels in 2-month-old WT *Sv129* males (**B**) and *Sv129* females (**C**) at different time points after osteocalcin injection. (**D**) Circulating aldosterone levels 2 hours after vehicle or osteocalcin injection at 1200 hours in 2-month-old male WT *Sv129* mice. (**E** and **F**) circulating aldosterone levels in *Sv129* male (**E**) and *Sv129* female (**F**) WT mice at different time points after osteocalcin injection. (**G**–**K**) Circulating osteocalcin (**G**), corticosterone (**H** and **J**), and aldosterone (**I** and **K**) levels in WT, *Esp_osb_^–/–^*, and *Esp_osb_^–/–^* *Ocn^+/–^* mice at 1800 hours. (**L**–**N**) Circulating osteocalcin (**L**), cortisol (**M**), and aldosterone (**N**) at different time points after vehicle or human osteocalcin injection at 1000 hours into rhesus monkeys. **S**tatistical analyses were conducted using 1-way ANOVA followed by Tukey’s post hoc test (**A**–**F**, **G**, **K**, **M**, and **N**) or a 2-tailed, unpaired *t* test (**H**–**J** and **L**). *P < 0.05. *n =* 6 or more each group for mice; *n =* 4 or more for rhesus monkeys.

We used gain-of-function experiments as a first approach to test whether osteocalcin affects GC biosynthesis, because these experiments allow one to address this question in diverse animal species. We found that, regardless of the source of osteocalcin, the time of the day, the sex, or the genetic background of the mice, a single injection of uncarboxylated, bioactive mouse osteocalcin (30 ng/g body weight) in 2-month-old (adult) WT mice increased circulating corticosterone 2 to 24 hours after injection by more than 2-fold (Figure 1, A–C, and Supplemental Figure 1, A and D; supplemental material available online with this article; https://doi.org/10.1172/JCI153752DS1). Since all these recombinant proteins were prepared using the same expression vector, the relatively modest differences in potency between these various preparations of recombinant osteocalcin likely reflect differences in the way the protein was prepared in distinct laboratories. Of note, the amplitude of this osteocalcin-induced rise in circulating corticosterone was similar to that seen 30 minutes after injecting a dose of ACTH (45 ng/g body weight) 20-fold lower than what is used for an ACTH test but equimolar to the dose of osteocalcin (Supplemental Figure 1B).

Unexpectedly, 2–24 hours after these osteocalcin injections into mice of either sex, of 2 different genetic backgrounds, at 2 different times of the day, we found that the levels of circulating aldosterone, the mineralocorticoid hormone synthesized by the zG of the adrenal cortex, increased to the same extent (Figure 1, D–F, and Supplemental Figure 1, C and E). Consistent with this delayed increase in circulating corticosterone and aldosterone, osteocalcin injections upregulated the expression of *Cyp11b1* and *Cyp11b2*, which encode key enzymes for the synthesis of corticosterone and aldosterone, respectively (Supplemental Figure 1F and ref. 5). Osteocalcin injections also enhanced the adrenal expression of *Mc2r*, the ACTH receptor, and *Agtr1a* and *Agtr1b,* the 2 angiotensin 2 receptors, but did not affect the expression of *Th*, which is needed to make catecholamine in the adrenal medulla (Supplemental Figure 1F).

To determine whether chronic elevation of circulating osteocalcin would also affect adrenal steroidogenesis, we analyzed *Esp_osb_^–/–^* mice, which represent a gain-of-osteocalcin-function model (Figure 1G and ref. 15). We found that circulating corticosterone and aldosterone levels were 35% and 25% higher, respectively, in adult *Esp_osb_^–/–^* mice than in control mice (Figure 1, H and I). Among all steroidogenic genes tested, only *Cyp11b1*, *Cyp11b2*, *Mc2r*, *Agtr1a*, and *Agtr1b* were expressed at higher levels in *Esp_osb_^–/–^* adrenal glands than in control adrenal glands, whereas hypothalamic *Crh* expression, circulating ACTH, and plasma renin activity were not significantly increased in *Esp_osb_*^–/–^ mice (Supplemental Figure 1, G–J). These observations suggest that osteocalcin signaled mainly in adrenal glands to promote adrenal steroidogenesis. We also observed that *Esp_osb_^–/–^* mice that lacked 1 allele of *Osteocalcin* had normal circulating osteocalcin, corticosterone, and aldosterone levels, indicating that osteocalcin favors adrenal steroidogenesis as a bone-derived molecule (Figure 1, G, J, and K).

To broaden the significance of these observations, we asked whether exogenous osteocalcin could also affect adrenal steroidogenesis in primates. We found that osteocalcin injections increased circulating cortisol and aldosterone in monkeys as well, albeit with different kinetics. Circulating cortisol levels rose 1 hour after injection, peaked at 2 hours, and returned to normal 6 hours after injection, whereas circulating aldosterone levels increased 3, 6, and even 24 hours after injection (Figure 1, L–N). On the other hand, circulating DHEA levels were not affected by these injections (Supplemental Figure 1K). Taken together, these data indicate that both acute and chronic elevation of circulating osteocalcin favored adrenal steroidogenesis in mice and nonhuman primates and selectively increased *Cyp11b1* and *Cyp11b2* expression in adrenal glands. Based on the higher expression of the receptors for these hormones, these observations suggest that osteocalcin may also increase ACTH and angiotensin 2 signaling in adrenal glands.

### Osteocalcin signaling on adrenal cells is necessary for adrenal steroidogenesis.

That osteocalcin can increase adrenal steroidogenesis raises an even more critical question: Is it a physiological regulator of this process? Given the number of organs involved in regulating the production of adrenal steroid hormones, addressing this question requires the identification of the receptor through which osteocalcin achieves this regulation and the organ(s) where this receptor is expressed.

**Figure 2:**
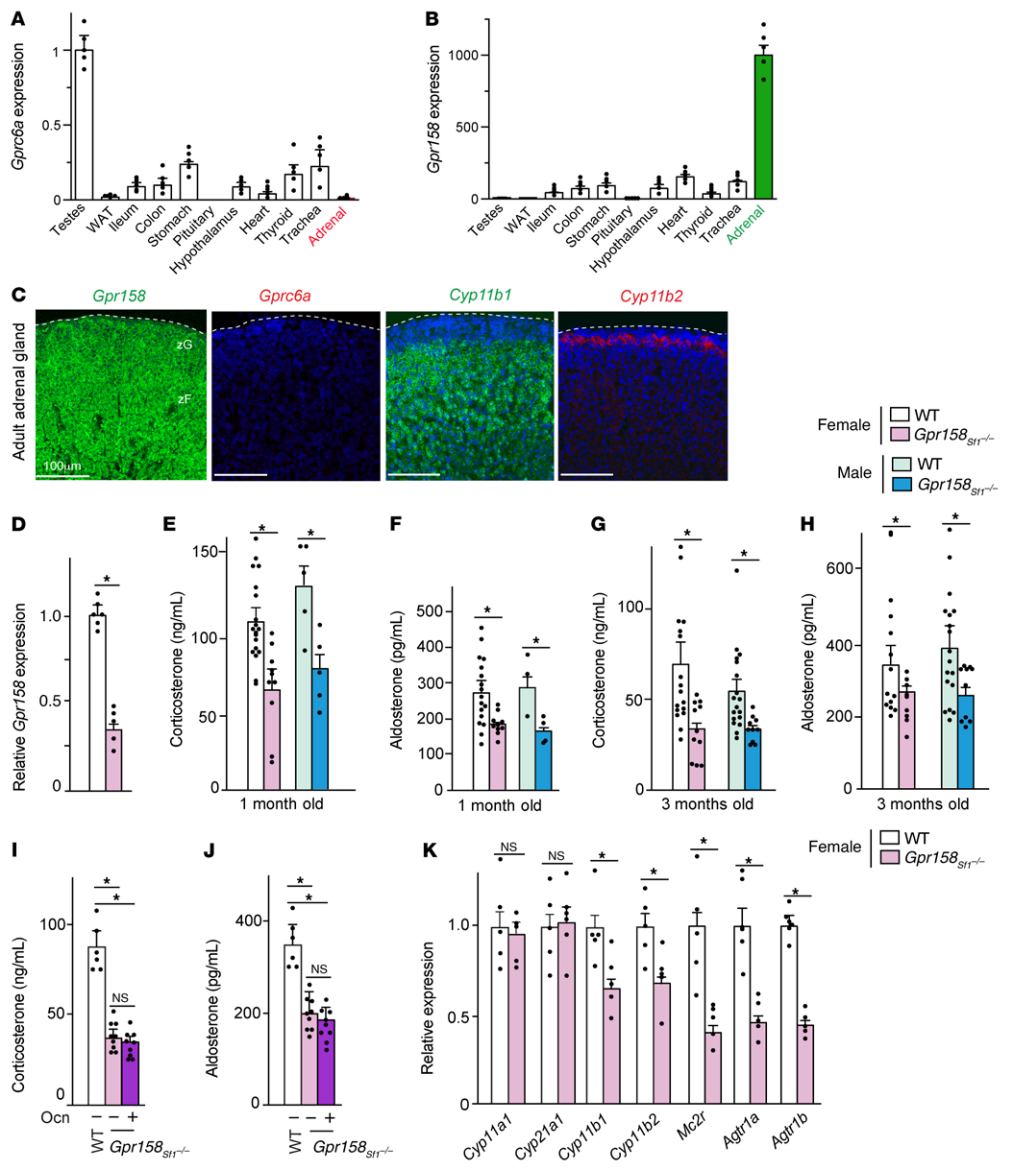
Osteocalcin signaling through Gpr158 in adrenal glands is necessary for adrenal steroidogenesis. (**A** and **B**) Expression of *Gprc6a* (**A**) and *Gpr158* (**B**) in different tissues from WT mice (qRT-PCR). (**C**) ISH analysis of *Gprc6a*, *Gpr158*, *Cyp11b1*, and *Cyp11b2* expression in WT adrenal glands. Scale bars: 100 μm. (**D**–**H**) *Gpr158* expression in adrenal glands (by qRT-PCR) (**D**), circulating corticosterone levels (**E**, 1-month-old and **G**, 3-month-old), and aldosterone levels in (**F**, 1-month-old and **H**, 3-month-old) female and male WT and *Gpr158_Sf1_^–/–^* mice. (**I** and **J**) Circulating corticosterone (**I**) and aldosterone (**J**) levels in 3-month-old WT and *Gpr158_Sf1_^–/–^* mice 2 hours after vehicle or osteocalcin injection. (**K**) Adrenal steroidogenic gene expression in WT and *Gpr158_Sf1_^–/–^* female mice. Statistical analyses were conducted using a 2-tailed, unpaired *t* test (**D**–**H** and **K**) or 1-way ANOVA followed by Tukey’s post hoc test (**I** and **J**). *P < 0.05. *n =* 6 or more mice per group.

The G protein–coupled receptor GPRC6A mediates most functions of osteocalcin in peripheral organs, however, its expression is undetectable in adrenal or pituitary glands (Figure 2A and ref. 13). In contrast, *Gpr158*, the receptor that transduces the osteocalcin signal in the brain, is expressed at least 1 order of magnitude higher in adrenal glands than in other peripheral tissues (Figure 2B and ref. 16). ISH analysis detected *Gpr158* expression in *Cyp11b1*- and *Cyp11b2-*expressing cells of the zF and zG, but not in cells of the adrenal medulla, pituitary gland, *Crh*-expressing neurons, or *Renin*-expressing cells of the kidney in WT mice (Figure 2C and Supplemental Figure 2, A–C). *Gprc6a* expression was undetectable in all these cell types (Figure 2C and Supplemental Figure 2, A and C).

**Figure 3:**
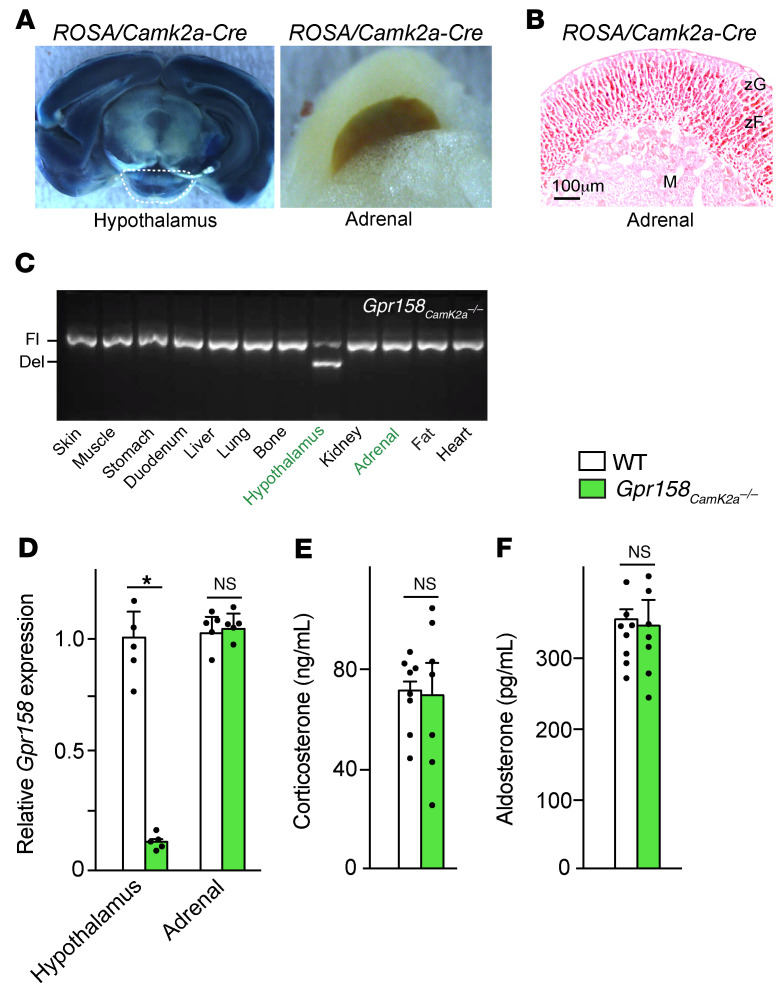
Neuronal deletion of *Gpr158* through *CamK2a-Cre* does not affect adrenal steroidogenesis. (**A**) β-Gal staining of a whole-mount mid-brain cross-section and adrenal gland from a 2-month-old *Camk2a-Cre^+^* mouse crossed with a ROSA reporter mouse. (**B**) Eosin- and β-gal–stained section of an adrenal gland from a 2-month-old *Camk2a-Cre^+^* mouse crossed with a ROSA reporter mouse. Scale bar: 100 μm. M, medulla. (**C**) Recombination analysis of genomic DNA in different tissues collected from *Gpr158_CamK2a_^–/–^* mice. Floxed (Fl) and deletion (Del) bands are indicated. (**D**–**F**) *Gpr158* expression in hypothalamus and adrenal glands (**D**) and circulating corticosterone (**E**) and aldosterone (**F**) levels in 3 month-old male WT and *Gpr158_Camk2a_^–/–^* mice. Statistical analyses were conducted using a 2-tailed, unpaired *t* test (**D**–**F**). *P < 0.05. *n =* 5 or more in each group.

Given this pattern of expression, we analyzed mice lacking *Gpr158* either in all cells (*Gpr158^–/–^*) or in adrenocortical cells, in *Crh-*expressing hypothalamic neurons, and pituitary corticotrope cells during development and postnatally (*Gpr158_Sf1_^–/–^*; ref. 17). We found that corticosterone and aldosterone adrenal content was significantly lower in 1-month-old *Gpr158^–/–^* mice than in WT littermates (Supplemental Figure 2, D and E). In *Gpr158_Sf1_^–/–^* mice, even though the gene deletion was not complete, we observed a 40%–50% reduction in circulating corticosterone levels and a 22%–34% reduction in circulating aldosterone levels compared with levels in control littermate mice, depending on their age and sex (Figure 2, D–H and Supplemental Figure 2, F and G). That exogenous osteocalcin failed to increase circulating corticosterone or aldosterone levels in *Gpr158_Sf1_^–/–^* mice as it did in control mice confirmed that osteocalcin signals through GPR158 to promote adrenal steroidogenesis (Figure 2, I and J). Circulating adrenal steroid hormones levels were normal in *Gprc6a^–/–^* mice (Supplemental Figure 2H). Expression of *Cyp11b1*, *Cyp11b2*, *Mc2r*, *Agtr1a*, and *Agtr1b* was significantly lower, whereas *Crh* expression, circulating ACTH levels, and plasma renin activity were either not decreased or were elevated in *Gpr158_Sf1_^–/–^* adrenal glands and mice compared with controls (Figure 2K and Supplemental Figure 2, I–K). These results are consistent with the fact that *Gpr158* was expressed in adrenocortical cells but not in *Crh*-expressing neurons, pituitary corticotropes, or *Renin*-expressing cells of the kidney (Figure 2C and Supplemental Figure 2, A and C). Furthermore, deletion of *Gpr158* from neurons, including *Crh-*expressing ones, but not from adrenal cells did not affect circulating corticosterone or aldosterone (Figure 3, A–F, and ref. 18). These results support the notion that osteocalcin signaling through GPR158 in cells of the adrenal cortex is necessary for adrenal steroidogenesis in the mouse.

### Embryonic osteocalcin signaling is necessary for proper adrenal steroidogenesis in adult offspring.

**Figure 4:**
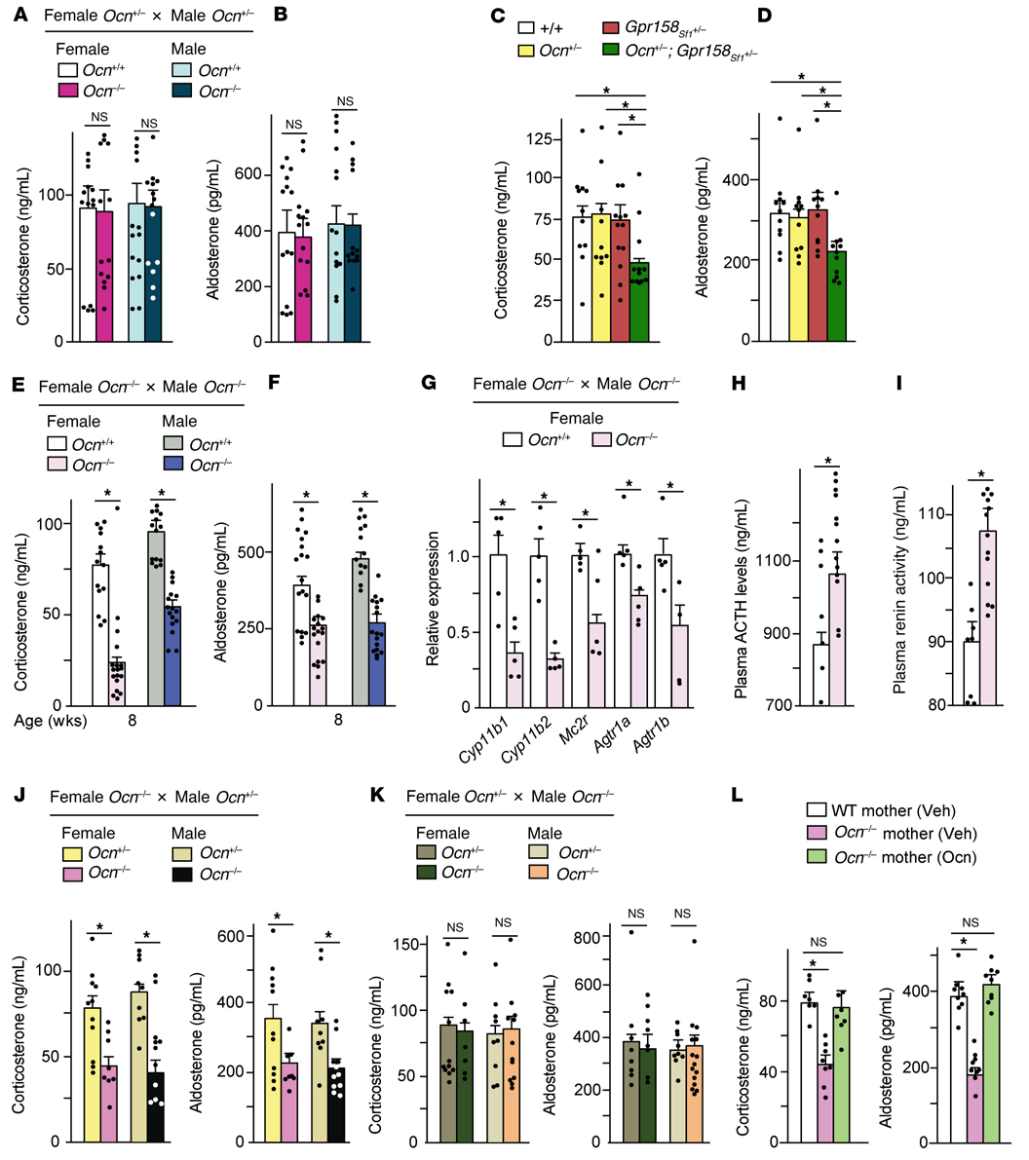
Embryonic osteocalcin promotes adrenal steroidogenesis and homeostasis in offspring. (**A** and **B**) Circulating corticosterone (**A**) and aldosterone (**B**) levels at 1800 hours in 2-month-old female and male *Ocn^–/–^* mice born from *Ocn^+/–^* parents and in WT littermates. (**C** and **D**) Circulating corticosterone (**C**) and aldosterone (**D**) levels at 1800 hours in 3-month-old WT, *Ocn^+/–^*, *Gpr158_Sf1_^+/–^*, and *Ocn^+/–^* *Gpr158_Sf1_^+/–^* mice born from *Ocn^+/–^* *Gpr158_Sf1_^+/–^* parents. (**E** and **F**) Circulating corticosterone (**E**) and aldosterone (**F**) levels at 1800 hours in 8-week-old *Ocn^+/+^* and *Ocn^–/–^* female and male mice born from *Ocn^+/+^* or *Ocn^–/–^* isogenic parents. (**G**–**I**) Adrenal steroidogenic gene expression (**G**), plasma ACTH levels (**H**), and plasma renin activity (**I**) in *Ocn^+/+^* and *Ocn^–/–^* female mice born from *Ocn^+/+^* or *Ocn^–/–^* isogenic parents. (**J** and **K**) Circulating corticosterone and aldosterone levels at 1800 hours in 2-month-old *Ocn^+/–^* and *Ocn^–/–^* female and male mice born from *Ocn^–/–^* (**J**) or *Ocn^+/–^* (**K**) mothers crossed with *Ocn^+/–^* or *Ocn^–/–^* fathers, respectively. (**L**) Circulating corticosterone and aldosterone levels in 2-month-old *Ocn^+/+^* and *Ocn^–/–^* mice born from *Ocn^+/+^* or *Ocn^–/–^* mothers that received either vehicle or osteocalcin (300 ng/day) from E14.5 until birth. In each panel, the parents are indicated on the top and progeny on the bottom. Statistical analyses were conducted using a 2-tailed, unpaired *t* test (**A**, **B**, and **E**–**K**) or 1-way ANOVA followed by Tukey’s post hoc test (**C**, **D**, and **L**). *P < 0.05. *n =* 10 or more in each group except for **G**–**I**: *n =* 5 or more.

The observations presented above inferred that adrenal steroidogenesis would be hampered in *Osteocalcin^–/–^* (*Ocn^–/–^*) mice. Surprisingly, however, for all adrenal parameters analyzed, adult *Ocn^–/–^* mice and WT littermates were indistinguishable (Figure 4, A and B). One possible explanation for this observation could be that in vivo osteocalcin is not the ligand of GPR158 that promotes adrenal steroidogenesis. Although this hypothesis cannot be excluded a priori, the fact that osteocalcin did not increase adrenal steroidogenesis in *Gpr158_Sf1_^–/–^* mice and the fact that compound heterozygous mice lacking 1 allele of *Osteocalcin* and 1 allele of *Gpr158* in adrenal cells exhibited a deficit in adrenal steroidogenesis, while single heterozygous mice did not, argue that osteocalcin is the endogenous ligand of *Gpr158* in adrenal glands (Figure 2, I and J, and Figure 4, C and D). Therefore, we considered as an alternative explanation for this conundrum the possibility that maternal or embryonic osteocalcin might affect adrenal development to such an extent that it would disrupt adrenal steroidogenesis postnatally.

In support of this hypothesis, we found that circulating corticosterone and aldosterone levels were significantly lower in 8-, 24-, and 52-week-old *Ocn^–/–^* mice of either sex born from a cross between *Ocn^–/–^* parents than in WT mice or *Ocn^–/–^* mice born from a cross between *Ocn^+/–^* parents (Figure 4, E and F, and Supplemental Figure 4, A and B). Expression of *Cyp11b1*, *Cyp11b2*, *Mc2r*, *Agtr1a*, and *Agtr1b* was also decreased in adrenal glands of *Ocn^–/–^* mice born from *Ocn^–/–^* parents compared with what was observed in adrenal glands from WT mice or *Ocn^–/–^* mice born from *Ocn^+/–^* mothers (Figure 4G and Supplemental Figure 4, C and D). In contrast, *Crh* expression, circulating ACTH, and plasma renin activity were higher in *Ocn^–/–^* mice born from *Ocn^–/–^* parents than in WT or *Ocn^–/–^* mice born from *Ocn^+/–^* parents (Figure 4, H and I, and Supplemental Figure 4, E and F). Hence, *Gpr158_Sf1_^–/–^* and *Ocn^–/–^* mice born from *Ocn^–/–^* parents exhibited identical phenotypic and molecular adrenal abnormalities. Of note, an injection of osteocalcin increased circulating corticosterone and aldosterone levels in *Ocn^–/–^* mice born from *Ocn^–/–^* mothers (Supplemental Figure 4G).

The contribution of each parent to the adrenal insufficiency phenotype observed in *Ocn^–/–^* mice born from *Ocn^–/–^* parents was determined by crossing *Ocn^+/–^* males with *Ocn^–/–^* females or *Ocn^–/–^* males with *Ocn^+/–^* females and measuring circulating adrenal steroid hormones in their adult *Ocn^–/–^* progeny. This experiment revealed that circulating corticosterone and aldosterone levels were normal in *Ocn^–/–^* mice born from *Ocn^+/–^* mothers, but significantly lower in those born from *Ocn^–/–^* mothers (Figure 4, J and K). Furthermore, osteocalcin was not detected in the blood of *Ocn^–/–^* pups born from and nursed by *Ocn^+/–^* mothers, indicating that no measurable quantity of maternal osteocalcin was transferred through lactation in the mouse (Supplemental Figure 4H).

The data presented above established that osteocalcin must be present in the embryo’s general circulation for adrenal steroidogenesis to occur normally postnatally but did not distinguish between a maternal or embryonic origin of this pool of osteocalcin. If maternal osteocalcin influences postnatal adrenal steroidogenesis, *Ocn^+/–^* mice born from *Ocn^–/–^* mothers should have low circulating adrenal steroid hormone levels. If, on the other hand, embryonic osteocalcin contributes to adrenal steroidogenesis, *Ocn^+/–^* mice born from *Ocn^–/–^* mothers should have normal circulating adrenal steroid hormones. We observed the latter, indicating that embryonic osteocalcin influences adrenal steroidogenesis postnatally (Figure 4, J and K). To define when embryonic osteocalcin signaling is needed to assure proper adrenal steroidogenesis postnatally, we crossed male and female *Ocn^–/–^* mice and injected pregnant *Ocn^–/–^* females once daily with osteocalcin (300 ng) from E14.5 until birth. These injections normalized circulating corticosterone and aldosterone levels in adult *Ocn^–/–^* progeny, indicating that embryonic osteocalcin signaling between E14.5 and birth exerts a lifelong influence on adrenal steroidogenesis in offspring (Figure 4L).

### Embryonic osteocalcin signaling enforces homeostasis in adult offspring.

If embryonic osteocalcin signaling influences adrenal steroidogenesis postnatally to such an extent, it should also affect physiological functions that are regulated by adrenal steroid and contribute in that way to the maintenance of organismal homeostasis. To test this hypothesis, we first analyzed blood pressure, a physiological function that is regulated by adrenal steroid hormones, in control and mutant mice (6).

**Figure 5:**
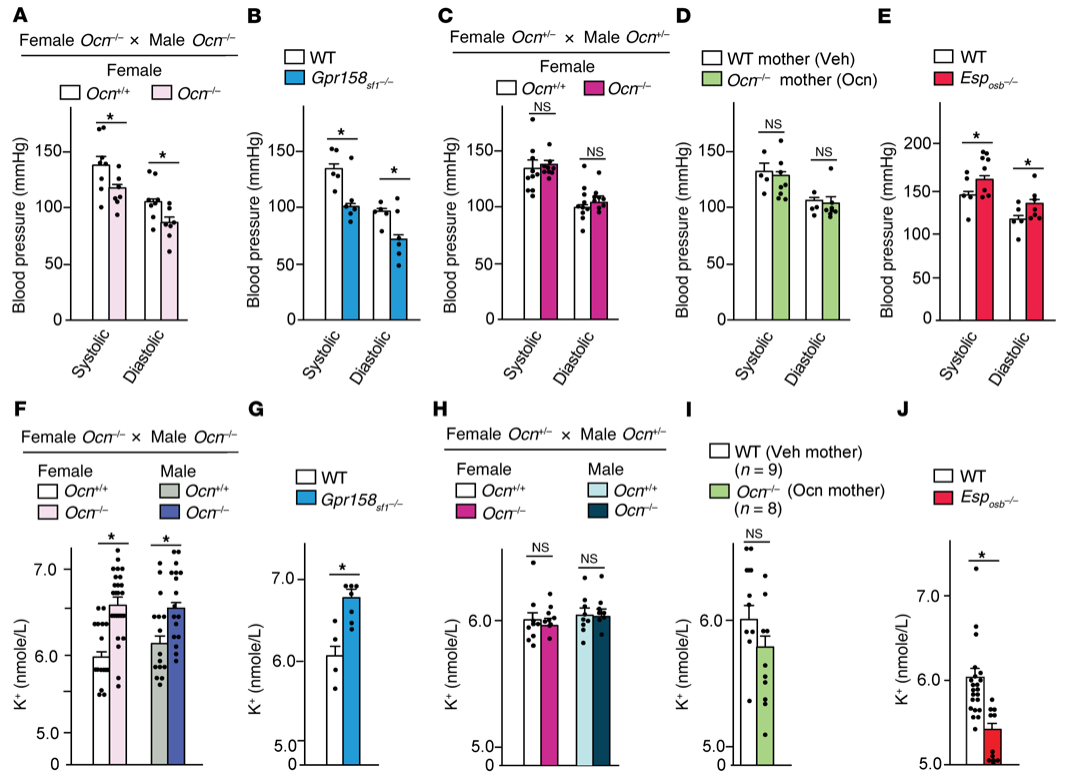
Embryonic osteocalcin promotes homeostasis in offspring. (**A**–**J**) Systolic and diastolic blood pressure and plasma K^+^ concentrations in 2-month-old *Ocn^+/+^* and *Ocn^–/–^* mice born from *Ocn^+/+^* or *Ocn^–/–^* isogenic parents (**A** and **F**); WT and *Gpr158_Sf1_ Ocn^–/–^* mice (**B** and **G**); *Ocn^+/+^* and *Ocn^–/–^* mice born from *Ocn^+/–^* parents (**C** and **H**); *Ocn^+/+^* and *Ocn^–/–^* offspring born from *Ocn^+/+^* or *Ocn^–/–^* mothers that received either vehicle (Veh mother) or osteocalcin (Ocn mother, 300 ng/day) from E14.5 until birth (**D** and **I**); and WT and *Esp_osb_^–/–^* mice (**E** and **J**). Statistical analyses were conducted using a 2-tailed, unpaired *t* test. *P < 0.05. *n =* 5 or more in each group.

We found that systolic and diastolic blood pressures were both significantly lower in adult *Gpr158_Sf1_^–/–^* and *Ocn^–/–^* mice born from *Ocn^–/–^* mothers than in control mice (Figure 5, A and B). Since the mothers of *Gpr158_Sf1_^–/–^* mice were *Gpr158^fl/fl^*, this observation established that it was embryonic osteocalcin signaling that was regulating blood pressure. In contrast, systolic and diastolic blood pressures were indistinguishable between WT mice and *Ocn^–/–^* mice born from *Ocn^+/–^* mothers, since osteocalcin from the mothers crossed the placenta and allowed normal adrenal development during embryogenesis (Figure 5C and ref. 19). The same was true in *Ocn^–/–^* mice born from *Ocn^–/–^* mothers injected once daily with osteocalcin from E14.5 to birth, even though they became deprived of osteocalcin postnatally (ref. 19 and Figure 5D). Conversely, in *Esp_osb_^–/–^* mice that had high circulating corticosterone and aldosterone levels, systolic and diastolic pressures were higher than in their littermate controls (Figure 5E). Since the mothers of *Esp_osb_^–/–^* mice were *Esp^fl/fl^,* this further confirmed that it was embryonic osteocalcin that was regulating in blood pressure in the adult mice (Figure 5E).

The second homeostatic parameter we analyzed was the blood K^+^ concentration, because it is regulated by aldosterone (6). We found that the blood K^+^ concentration was significantly higher in *Gpr158_Sf1_^–/–^* and *Ocn^–/–^* mice born from *Ocn^–/–^* mothers than in WT mice, *Ocn^–/–^* mice born from *Ocn^+/–^* mothers, or *Ocn^–/–^* mice born *Ocn^–/–^* mothers that had received osteocalcin injections daily from E14.5 to birth (Figure 5, F–I). Conversely, the blood K^+^ concentration was lower in *Esp_osb_^–/–^* mice that had high circulating osteocalcin and aldosterone levels (Figure 5J).

The last homeostatic function we analyzed was the elevation of circulating corticosterone that is triggered by the acute stress response. We observed that, following an exposure to 2,3,5-trimethyl-3-thiazoline (TMT) as a stressor, circulating corticosterone levels rose to significantly lower levels in *Ocn^–/–^* mice born from *Ocn*^–/–^ mothers than in *Ocn^–/–^* mice born from *Ocn*^+/–^ mothers (ref. 20 and Supplemental Figure 5A). Taken together, these data indicate that, through its regulation of adrenal steroidogenesis, embryonic osteocalcin signaling affected several important physiological functions and contributed to the maintenance of organismal homeostasis in adult offspring. Importantly, none of these physiological functions were affected by the absence of osteocalcin postnatally (Figure 5, C and H), since they were normal in *Ocn^–/–^* mice born from *Ocn^+/–^* mothers that received maternal osteocalcin, which crosses the placenta during pregnancy (19).

### Embryonic osteocalcin signaling promotes adrenal cell proliferation during development and affects lifelong adrenal growth.

**Figure 6:**
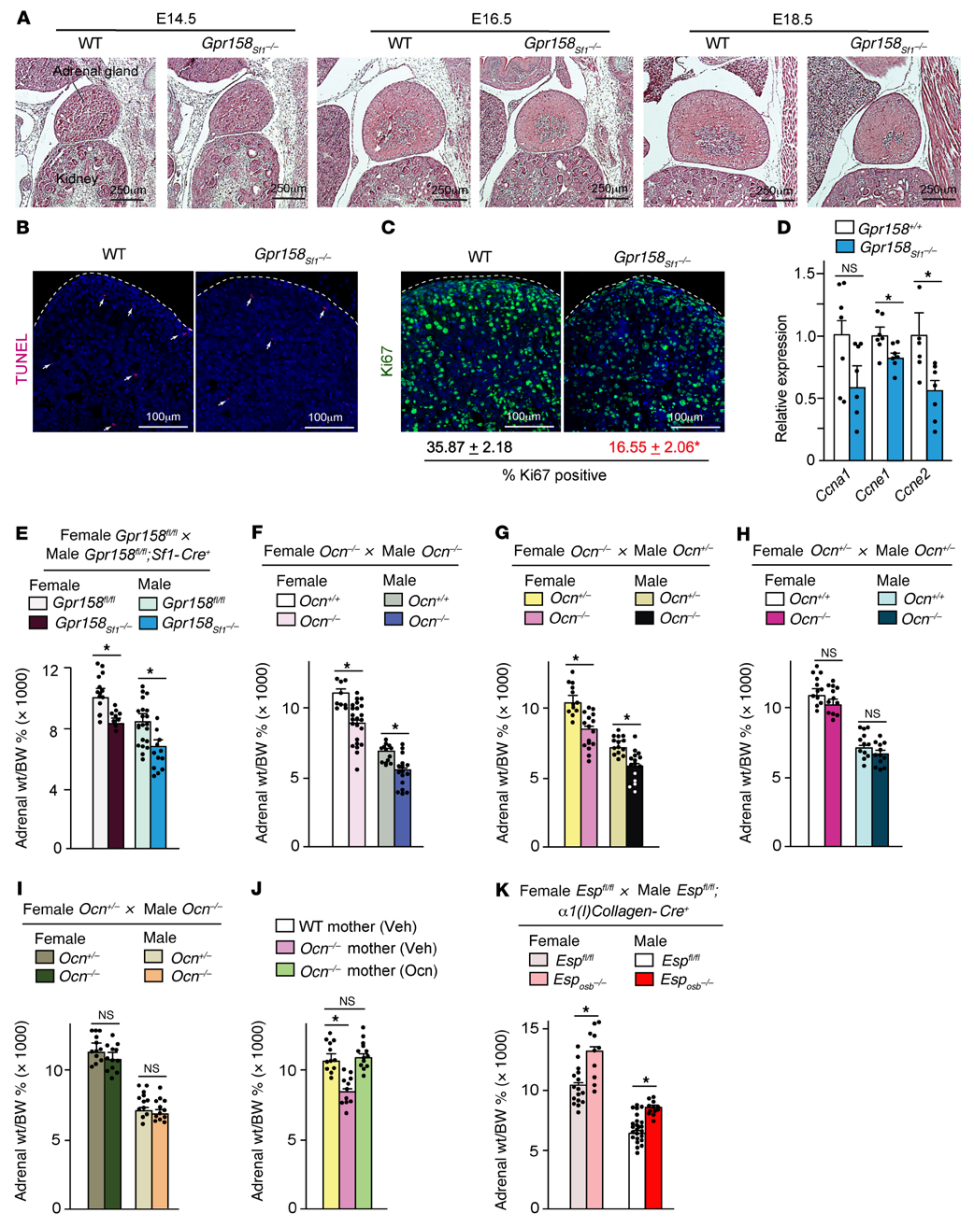
Embryonic osteocalcin signaling in adrenal glands promotes cell proliferation during development. (**A**) H&E-stained sections of adrenal glands of E14.5, E16.5, and E18.5 WT and *Gpr158_Sf1_^–/–^* embryos. Scale bars: 250 μm. (**B** and **C**) TUNEL staining showing apoptosis (**B**) and Ki67 staining showing proliferation (**C**) in E18.5 adrenal glands from WT and *Gpr158_Sf1_^–/–^* embryos. Scale bars: 100 μm. (**D**) *Cyclin* gene expression in adrenal glands from WT and *Gpr158_Sf1_^–/–^* newborn mice. (**E**–**K**) Adrenal gland per body weight percentage (Adrenal wt/BW %) for mice of the indicated genotypes and crosses. In each panel, parents are indicated on the top and progeny on the bottom. Statistical analyses were conducted using a 2-tailed, unpaired *t* test (**D**–**I** and **K**) or 1-way ANOVA followed by Tukey’s post hoc test (**J**). *P < 0.05. *n =* 10 or more in each group (**E**–**K**); *n =* 5 or more in each group (**A**–**C**).

How could osteocalcin signaling during development affect adrenal steroidogenesis so significantly in the adult offspring? To begin addressing this fundamental question, we studied adrenal cell proliferation in embryos and newborn mice lacking osteocalcin signaling in adrenal cells. Although the adrenal glands of *Gpr158_Sf1_^–/–^* E14.5 embryos were indistinguishable from those of control embryos, they were markedly smaller in E16.5 and E18.5 *Gpr158_Sf1_^–/–^* embryos compared with controls (Figure 6A). This small adrenal gland size in *Gpr158_Sf1_^–/–^* embryos could not be explained by low circulating levels of corticosterone, since the mothers of *Gpr158_Sf1_^–/–^* mice were *Gpr158^fl/fl^* and have normal circulating corticosterone levels (Figure 2, E and F).

Thus, to explain this delay in adrenal gland growth in *Gpr158_Sf1_^–/–^* embryos, we studied adrenal cell apoptosis and proliferation. While apoptosis as measured by a TUNEL assay was not different in control or mutant adrenal glands at any developmental stage analyzed, cell proliferation, assessed by Ki67 staining, was decreased by approximately 2-fold in adrenal glands of E18.5 *Gpr158_Sf1_^–/–^* embryos compared with those of control littermates (Figure 6, B and C). This decrease in cell proliferation was specific to adrenal glands, as Ki67 staining was identical in the livers of E18.5 *Gpr158_Sf1_^–/–^* and control embryos (Supplemental Figure 6A). In support of the notion that adrenal cell proliferation is regulated by osteocalcin signaling during development, we found that expression of Cyclin A1 (*Ccna1*), Cyclin E1 (*Ccne1)*, and Cyclin E 2 (*Ccne2*) was markedly decreased in *Gpr158_Sf1_^–/–^* adrenal glands compared with expression in control glands at birth (Figure 6D). This developmental deficit in adrenal cell proliferation had postnatal consequences, since adrenal gland weight remained significantly lower in *Gpr158_Sf1_^–/–^* than in control mice during adulthood (Figure 6E). Adrenal gland weight and cell proliferation were also lower in adult *Ocn^–/–^* mice born from *Ocn^–/–^* mothers than in control mice but were normal in adult *Ocn^–/–^* mice born from *Ocn^+/–^* mothers or *Ocn^–/–^* mothers that had received daily osteocalcin injections from E14.5 until birth (Figure 6, F–J, and Supplemental Figure 6B). Conversely, adrenal gland weight was significantly higher in *Esp_osb_^–/–^* mice than in control mice (Figure 6K). Thus, osteocalcin signaling during embryonic development is necessary for adrenal cell proliferation and, as a result, determines adrenal gland growth and size throughout life.

### Embryonic osteocalcin controls the cascade of adrenal steroidogenic cell differentiation.

**Figure 7:**
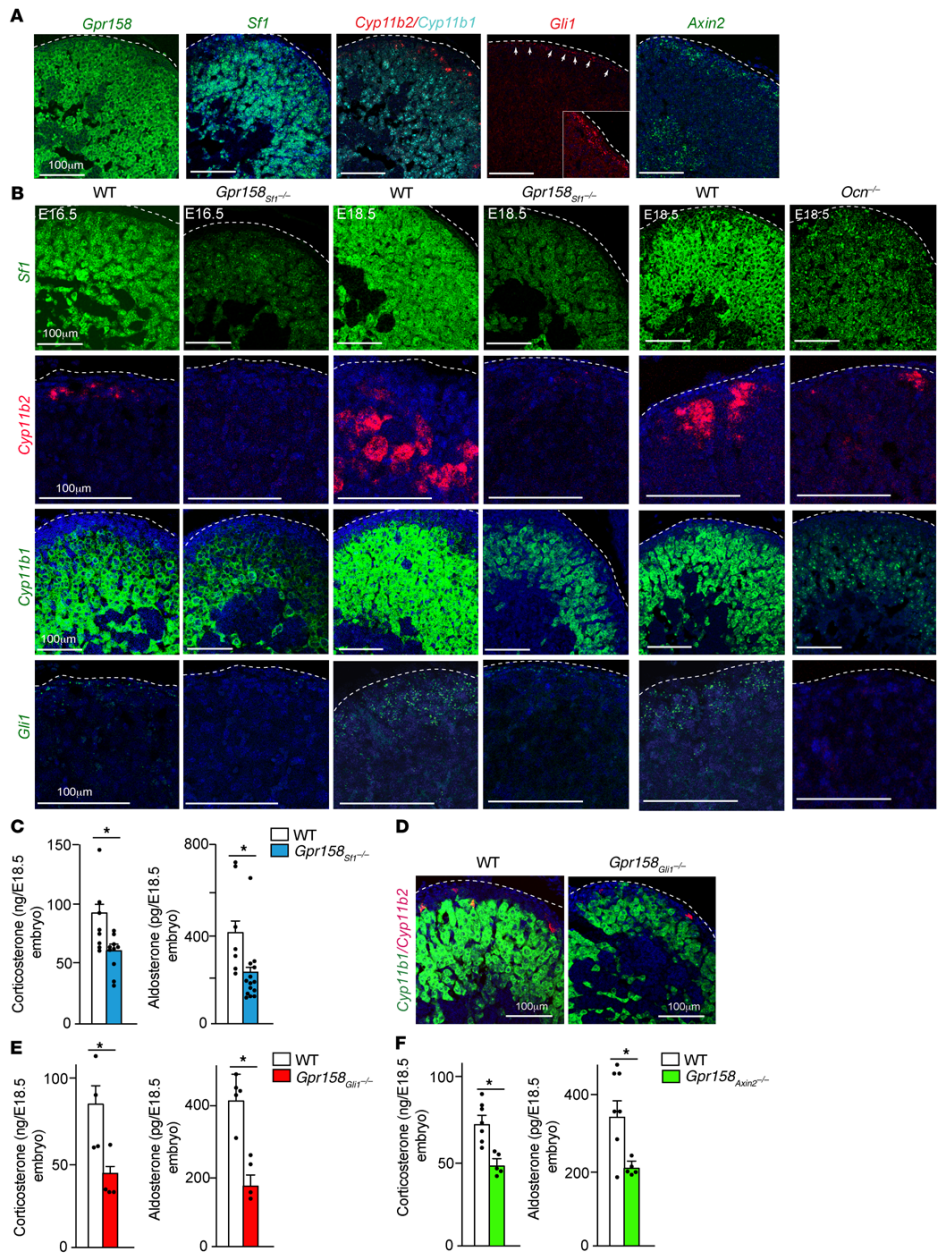
Embryonic osteocalcin signaling in adrenal glands establishes the steroidogenic program during development. (**A**) ISH analysis of adrenal *Gpr158*, *Sf1*, *Cyp11b1*, *Cyp11b2*, *Gli1*, and *Axin2* expression in E18.5 WT embryos. Scale bars: 100 μm. (**B**) ISH analysis of adrenal *Sf1*, *Cyp11b2*, *Cyp11b1*, and *Gli1* expression in E16.5 and E18.5 WT and *Gpr158_Sf1_^–/–^* embryos and E18.5 WT and *Ocn^–/–^* embryos. Scale bars: 100 μm. (**C**) Intra-adrenal content of corticosterone and aldosterone in E18.5 WT and *Gpr158_Sf1_^–/–^* embryos. (**D**) ISH analysis of *Cyp11b2* and *Cyp11b1* expression in E18.5 WT and *Gpr158_Gli1_^–/–^* embryos. Scale bars: 100 μm. (**E**) Intra-adrenal content of corticosterone and aldosterone in E18.5 WT and *Gpr158_Gli1_^–/–^* (**E**) and *Gpr158_Axin2_^–/–^* (**F**) embryos. Statistical analyses were conducted using a 2-tailed, unpaired *t* test (**C**, **E**, and **F**). *P < 0.05. *n =* 5 or more in each group of embryos or mice (**C,** **E,** and **F**); *n =* 3 or more for the ISH analysis (**A,** **B,** and **D**).

To elucidate how osteocalcin signaling during development could affect adrenal steroidogenesis throughout life, we also analyzed the expression of genes implicated in adrenal development in WT (Figure 7A) and mutant embryos lacking either *Gpr158* expression in adrenal glands or *Osteocalcin* expression (Figure 7B).

*Sf1* expression marks both fetal adrenal cells and steroidogenic ones in the developing adrenal glands (5, 21). Although adrenal *Sf1* expression was similar in E14.5 *Gpr158_Sf1_^–/–^* and control littermate embryos, it was markedly decreased in adrenal glands of E16.5 and 18.5 *Gpr158_Sf1_^–/–^* embryos and in those of E18.5 *Ocn^–/–^* embryos carried by *Ocn^–/–^* mothers compared with adrenal glands of control embryos; the same was true for the expression of *Cyp11b2* and *Cyp11b1* in the zG and zF, respectively (Figure 7B and Supplemental Figure 7, A and B). Accordingly, intra-adrenal content of aldosterone and corticosterone was low in E18.5 *Gpr158_Sf1_^–/–^* embryos (Figure 7C). In contrast, *Cyp11b1* and *Cyp11b2* expression in adrenal glands was identical in *Ocn^–/–^* newborn pups born from *Ocn^+/–^* mothers and in those born from WT mice (Supplemental Figure 7C).

*Sf1*-expressing fetal adrenal cells give rise to adrenocortical progenitor cells that reside in the adrenal capsule and express *Gli1* (1, 5). That *Gli1* expression was dramatically reduced in adrenal glands of E16.5 and 18.5 *Gpr158_sf1_^–/–^* embryos indicated that osteocalcin signaling in *Sf1*-positive fetal adrenal cells was also needed for the generation of *Gli1*-positive adrenocortical progenitor cells (Figure 7B). In support of this notion, inactivation of *Gpr158* in *Gli1*-positive adrenocortical progenitor cells dramatically decreased *Cyp11b1* and *Cyp11b2* expression in cells of the zG and zF and the intra-adrenal content of corticosterone and aldosterone in E18.5 *Gpr158_Gli1_^–/–^* embryos (Figure 7, D and E, and Supplemental Figure 7, D and E).

Last, we asked whether osteocalcin signaling contributes to the differentiation of cells of the zG into cells of the zF. Wnt signaling favors the renewal and differentiation of zG cells into zF cells, and *Axin2* is often used as a marker of Wnt signaling in adrenal cells (4, 22, 23). That the intra-adrenal content of aldosterone and corticosterone was decreased by 45 and 33%, respectively, in E18.5 *Gpr158_Axin2_^–/–^* embryos compared with control ones (Figure 7F) indicated that osteocalcin signaling in *Axin2*-positive cells is necessary for the differentiation of zG cells into zF cells. Taken together, these gene expression and functional analyses in various mutant embryos indicated that osteocalcin signaling was needed after E14.5 for the entire cascade of adrenal steroidogenic cell differentiation including *Sf1* expression in fetal adrenal cells, the generation of *Gli1*-positive adrenocortical progenitor cells, and the differentiation of these progenitor cells into steroidogenic cells of the zG and zF.

### Osteocalcin can induce adrenal growth and steroidogenesis in the absence of ACTH signaling.

**Figure 8:**
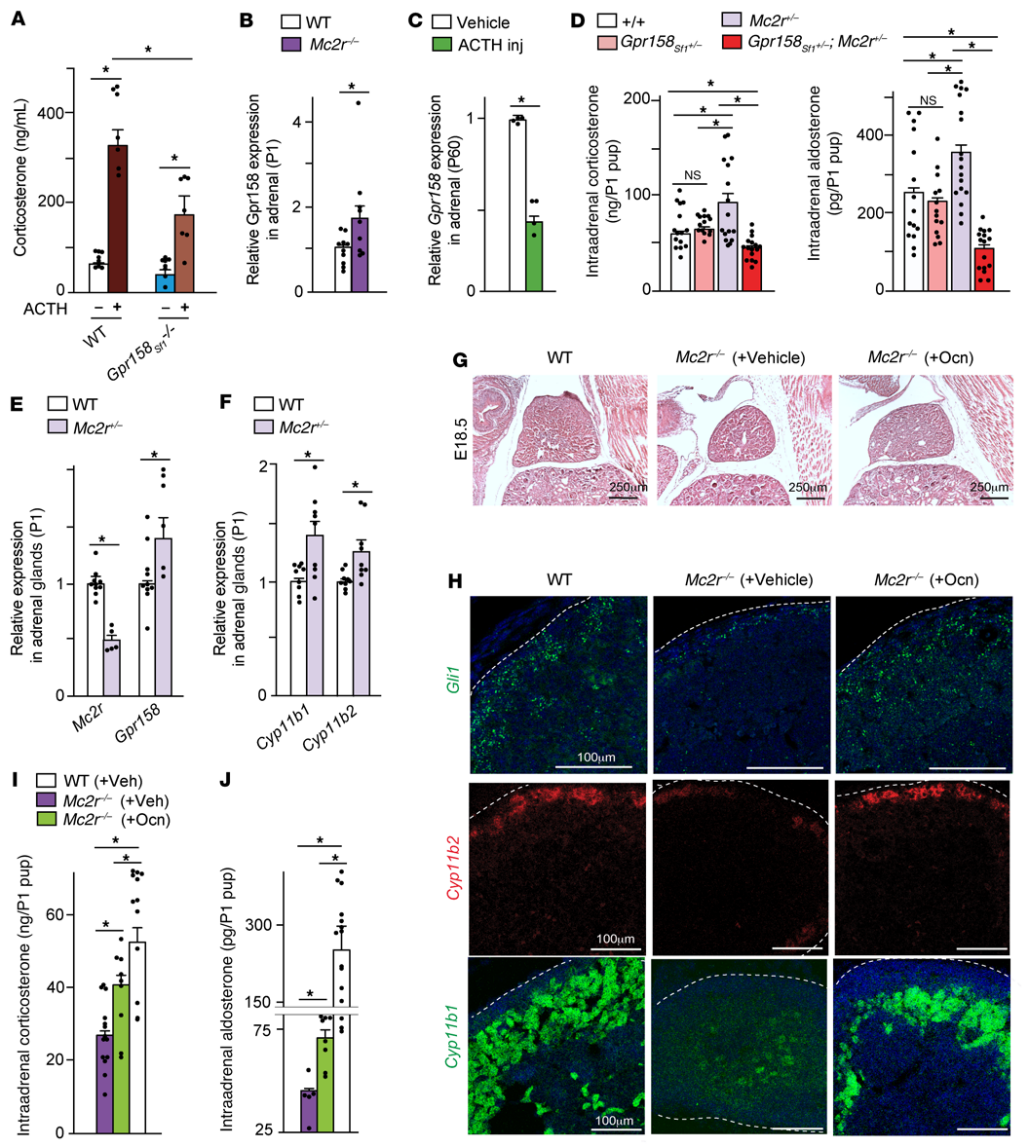
Osteocalcin induces adrenal steroidogenesis and growth in the absence of ACTH signaling. (**A**) Circulating corticosterone levels following an acute ACTH challenge at 1000 hours in adult WT and *Gpr158_sf1_^–/–^* mice born from a *Gpr158^fl/fl^* female mouse crossed with a *Gpr158^fl/fl^* *Sf1-Cre^+^* male mouse. (**B**) *Gpr158* expression in adrenal glands of WT and *Mc2r^–/–^* newborn mice. (**C**) Adrenal *Gpr158* expression in WT mice 2 hours after ACTH challenge. (**D**) Intra-adrenal content of corticosterone and aldosterone in P1 WT, *Mc2r^+/–^*, *Gpr158_Sf1_^+/–^*, and *Mc2r^+/–^* *Gpr158_Sf1_^+/–^* mice born from *Mc2r^+/–^* *Gpr158_Sf1_^+/–^* parents. (**E** and **F**) Adrenal *Mc2r* (**E**), *Gpr158* (**E**), *Cyp11b1*, and *Cyp11b2* (**F**) expression in WT and *Mc2r^+/–^* newborn mice. (**G** and **H**) H&E-stained sections of adrenal glands (**G**) and ISH analysis of adrenal *Gli1*, *Cyp11b2*, and *Cyp11b1* expression (**H**) in E18.5 WT and *Mc2r^–/–^* embryos collected from *Mc2r^+/–^* mothers that received either vehicle or osteocalcin (300 ng/day) from E10.5 to E18.5. Scale bars: 250 μm (**G**) and 100 μm (**H**). (**I** and **J**) Intra-adrenal content of corticosterone (**I**) and aldosterone (**J**) in WT and *Mc2r^–/–^* newborn mice born from *Mc2r^+/–^* mothers that received vehicle or osteocalcin (300 ng/day) from E10.5 until birth. Statistical analyses were conducted using 1-way ANOVA followed by Tukey’s post hoc test (**A**, **D**, **I**, and **J**) or 2-tailed, unpaired *t* test (**B**, **C**, **E**, and **F**). *P < 0.05. *n =* 6 or more embryos or offspring in each group.

In addition to enhancing adrenal cell proliferation, cell differentiation, and steroidogenesis, osteocalcin signaling favors *Mc2r* expression in adrenal glands (Figure 2K and Supplemental Figure 1, F and G). This explains why there was a significantly lower rise in circulating corticosterone levels in *Gpr158_Sf1_^–/–^* mice that had lower *Mc2r* expression than in control mice during an ACTH test (Figure 8A). This positive regulation of ACTH signaling by osteocalcin raises the question as to whether osteocalcin favors adrenal growth and steroidogenesis indirectly by promoting ACTH signaling, or directly and independently of its regulation of ACTH signaling in adrenal glands. To address this question, we took advantage of the unexpected observation that *Gpr158* expression was increased in the adrenal glands of *Mc2r^–/–^* newborn mice (Figure 8B). In agreement with this observation suggesting that ACTH inhibits Gpr158 expression, ACTH signaling through Mc2r decreased *Gpr158* expression in adrenal glands (Figure 8C).

We and others have observed that, despite their 50% decrease in adrenal *Mc2r* expression, *Mc2r^+/–^* mice exhibited a paradoxical increase in intra-adrenal content of steroid hormones (Figure 8D and ref. 24). We tested the hypothesis that this steroid hormone increase was secondary to an increase in osteocalcin signaling. In support of this hypothesis, we found that the HPA axis inhibited *Gpr158* expression, since the expression of this latter gene and of *Cyp11b1* and *Cyp11b2* was markedly higher in the adrenal glands of *Mc2r^+/–^* newborn mice than in those of their littermate controls (Figure 8, E and F). The fact that compound heterozygous *Gpr158_Sf1_^+/–^ Mc2r^+/–^* newborn mice had low intra-adrenal content of steroid hormones, but individual heterozygous mice did not, suggested that *Mc2r^+/–^* mice had enhanced adrenal steroidogenesis because of an increase in osteocalcin signaling (Figure 8D).

To establish whether this was indeed the case, we crossed male and female *Mc2r*^+/–^ mice and administered osteocalcin to pregnant females once a day (300 ng/day, i.p.,) from E10.5 until E18.5 or birth. We then analyzed *Mc2r^–/–^* E18.5 embryos and newborn mice. We observed that the adrenal glands of E18.5 *Mc2r^–/–^* embryos carried by osteocalcin-injected *Mc2r^+/–^* mothers were 30% larger than those of E18.5 *Mc2r^–/–^* embryos carried by vehicle-injected *Mc2r^+/–^* mothers (Figure 8G). The numbers of *Gli1*-positive adrenocortical progenitor cells and *Cyp11b1*- and *Cyp11b2*-positive steroidogenic cells were substantially higher in E18.5 *Mc2r^–/–^* embryos carried by osteocalcin-treated *Mc2r^+/–^* mothers than in those carried by vehicle-injected *Mc2r^+/–^* mothers (Figure 8H). When we conducted this analysis in newborn mice, we found that 70% of *Mc2r^–/–^* pups born from vehicle-injected *Mc2r^+/–^* mothers died within 12 hours of delivery (Table 1). In contrast, 66% of *Mc2r*^–/–^ pups born from osteocalcin-injected *Mc2r^+/–^* mothers were still alive 36 hours after birth (Table 1); these latter pups had 50% and 80% higher adrenal corticosterone and aldosterone content, respectively, than did *Mc2r^–/–^* newborn mice born from vehicle-injected *Mc2r^+/–^* mothers (Figure 8, I and J). To provide molecular evidence that exogenous osteocalcin could signal in adrenal glands even in the complete absence of *Mc2r* expression, we took advantage of the finding that the accumulation of phosphorylated CREB (p-CREB) in adrenal glands, the active form of this transcription factor (25), was decreased in the absence of osteocalcin signaling (data not shown). We then asked whether exogenous osteocalcin could restore, even partially, the accumulation of p-CREB in the adrenal glands of *Mc2r^–/–^* mice. As anticipated, we found that p-CREB was abundantly present in WT adrenal glands and absent in the adrenal glands of *Mc2r^–/–^* newborn mice whose mothers were treated with vehicle. In contrast, p-CREB accumulation was restored to near WT levels in the adrenal glands of *Mc2r^–/–^* newborn mice whose mothers were injected daily with osteocalcin during their pregnancy (Supplemental Figure 8A). These data show that until birth, osteocalcin can signal in adrenal glands, induce adrenal growth, and promote adrenal steroidogenesis in the absence of a functional HPA axis.

## Discussion

This study was initiated to address a lingering question of bone biology: Why do GC hormones regulate *Osteocalcin* expression? Our working hypothesis was that the inhibition of *Osteocalcin* expression by GC infers that osteocalcin regulates GC biosynthesis. Gain- and loss-of-function experiments performed in rodents and/or primates established that this was indeed the case. Embryo-derived (embryonic) osteocalcin signaling between E14.5 and birth during mouse embryogenesis is necessary, regardless of the presence or absence of a functional HPA axis, for fetal adrenal cell maintenance, differentiation of fetal adrenal cells into adrenocortical progenitor cells, and differentiation of these latter cells into steroidogenic cells. As a result, in adult offspring, embryonic osteocalcin signaling affected adrenal growth, adrenal steroidogenesis, and aspects of organismal homeostasis that postnatal osteocalcin did not affect (Figure 7). Hence, an embryonic hormone determines organismal homeostasis throughout life. Along with other work, this study also identified bone as a major regulator of steroidogenesis in the kidneys, testes, and adrenal glands (26–28).

**Figure 9:**
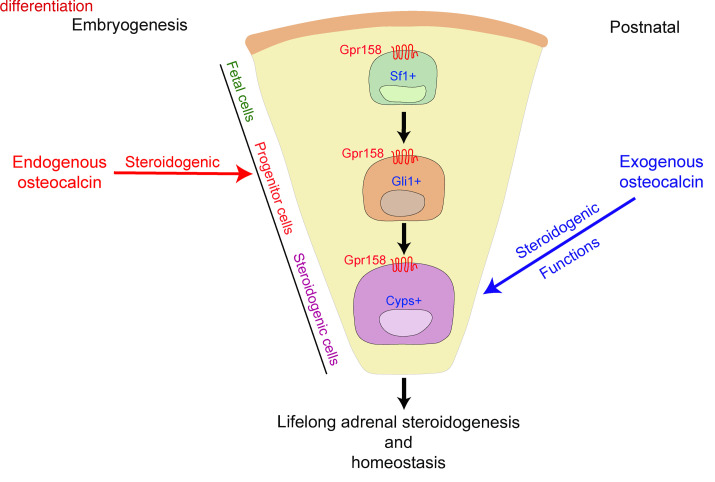
Model of the regulation of adrenal steroidogenesis and postnatal homeostasis by osteocalcin. Embryonic osteocalcin signaling in the developing adrenal gland through Gpr158 is necessary for the differentiation of fetal, progenitor, and steroidogenic adrenal cells as well as for the proliferation of these cells. This affects lifelong adrenal growth and steroidogenesis and homeostasis in the offspring. Postnatally, exogenous osteocalcin can enhance steroidogenic functions in rodents and nonhuman primates.

Besides establishing that osteocalcin was sufficient to increase GC biosynthesis in rodents and nonhuman primates, our gain-of-function experiments provided several additional insights (Figure 9). First, raising circulating osteocalcin levels increased to a similar extent circulating corticosterone and aldosterone levels, suggesting that osteocalcin regulates the biosynthesis of both adrenal steroid hormones (1). Second, adrenal glands were larger in mice with a chronic increase in circulating osteocalcin, implying that osteocalcin might promote adrenal growth. Last, by showing that osteocalcin upregulated the expression of genes encoding key enzymes in adrenal steroidogenesis, but not circulating ACTH levels or plasma renin activity, these experiments suggested that osteocalcin enhanced adrenal steroidogenesis principally by signaling in adrenal glands, although we cannot formally exclude the possibility that it also acts posttranscriptionally.

All these contentions were verified through loss-of-function experiments. Adult mice lacking *Gpr158*, the osteocalcin receptor expressed in cells of the adrenal cortex, experienced low circulating levels of both corticosterone and aldosterone and reduced adrenal gland weight compared with control mice. The same was true for compound heterozygous mice lacking 1 allele of *Osteocalcin* and 1 allele of *Gpr158*. Moreover, the adrenal insufficiency phenotype of *Gpr158_Sf1_^–/–^* mice could not be corrected by osteocalcin injections. Several arguments indicate that osteocalcin signals preferentially, if not only, in the adrenal cortex, where *Gpr158* is expressed. First, we could not detect *Gpr158* expression in *Crh*-expressing neurons, in cells of the pituitary glands, or in *Renin*-expressing cells of the kidney. Second, expression of both *Cyp11b1* and *Cyp11b2* was low in *Gpr158_Sf1_^–/–^* adrenal glands, while we detected no decrease in *Crh* expression, circulating levels of ACTH, or plasma renin activity in *Gpr158_Sf1_^–/–^* mice. Third, the deletion of *Gpr158* in neurons including *Crh-*expressing ones, did not affect adrenal steroidogenesis. There were, however, differences between the endocrine profile of *Ocn*^–/–^mice born from *Ocn^–/–^* mothers and of *Gpr158_Sf1_^–/–^* mice. For instance, plasma ACTH levels and plasma renin activity were higher in *Ocn^–/–^* mice than in *Gpr158_Sf1_^–/–^* mice compared with their respective controls (compare Figure 4, H and I, and Supplemental Figure 2, J and K). We believe this difference reflects the fact that the deletion of *Gpr158* was incomplete in this model of cell-specific gene deletion.

The lifelong regulation of adrenal growth and steroidogenesis by osteocalcin signaling is a result of these developmental functions. Indeed, adult *Ocn^–/–^* mice born from *Ocn^–/–^* mothers had lower circulating adrenal steroid hormone levels and smaller adrenal glands than do WT mice, whereas *Ocn^–/–^* mice born from *Ocn^+/–^* parents that became deprived of osteocalcin only after birth, and *Ocn^–/–^* mice born from *Ocn^–/–^* mothers that had received daily injections of osteocalcin from E14.5 until birth, did not. Two additional pieces of evidence indicated that it was embryonic osteocalcin that determined postnatal adrenal growth and steroidogenesis in the offspring. First, adrenal growth and steroidogenesis were increased in *Esp_osb_^–/–^* mice born from *Esp^fl/fl^* mothers that had normal osteocalcin levels. Second, adrenal growth and steroidogenesis were normal in *Ocn^+/–^* mice born from *Ocn^–/–^* mothers. The importance of the lifelong influence of embryonic osteocalcin on adrenal steroidogenesis is best illustrated by the fact that adrenal steroidogenesis, blood pressure, blood K^+^ concentration, the ability to increase circulating corticosterone during an acute stress response, and adrenal growth were all altered in adult *Ocn^–/–^*mice born from *Ocn^–/–^* mothers. Although exogenous osteocalcin could increase adrenal steroidogenesis after birth, postnatally, endogenous osteocalcin did not affect either adrenal steroidogenesis or homeostasis in any measurable manner. We found that embryonic osteocalcin signaling affected postnatal adrenal growth and steroidogenesis throughout life, in large part because it determined between E14.5 and birth the expression of *Sf1* in fetal adrenal cells, the generation and proliferation of *Gli1*- and *Axin2*-positive nonsteroidogenic adrenocortical progenitor cells in the subcapsular zone of the developing adrenal gland, and the generation of adrenal steroidogenic cells that express *Cyp11b1* or *Cyp11b2*. These observations raise the question of embryonic osteocalcin’s contribution to other functions of this hormone.

The available evidence not only indicated that osteocalcin signaling was necessary for adrenal development and steroidogenic functions, but that this regulation was active and could sustain some level of adrenal steroid hormone production even when the HPA axis was disrupted. The evidence supporting this statement is that adrenal steroid hormones were elevated in *Mc2r^+/–^* mice and that exogenous osteocalcin could rescue the phenotype of *Mc2r^–/–^* embryos and newborn mice. These findings do not exclude the possibility that osteocalcin and ACTH signaling may regulate each other. For instance, osteocalcin is necessary for normal *Mc2r* expression levels in adrenal glands through molecular pathways that may include IP production, as is the case in hippocampal neurons and also CREB-dependent signaling, as shown in Supplemental Figure 8 (16). In contrast, *Mc2r* expression inhibited *Gpr158* expression in adrenal glands through currently unknown molecular mechanisms.

Several explanations may account for why osteocalcin signaling in adrenal glands was dispensable for adrenal growth and steroidogenesis postnatally. It is possible that ACTH signaling hampered osteocalcin signaling in adrenal glands more strongly in adult mice than in embryos. This explanation is consistent with the significant increase in circulating ACTH and even more so in *Mc2r* expression in adult animals compared with embryos (29). The decrease in circulating osteocalcin levels in adult mice may also partly explain why the postnatal absence of osteocalcin did not affect adrenal growth or steroidogenesis. We believe, however, that the most likely explanation is that osteocalcin signaling is necessary for adrenal cell differentiation during embryogenesis but not for steroidogenic gene expression per se. In agreement with this contention, when adrenal cell differentiation occurred normally during embryogenesis, as was the case in *Ocn*^–/–^ mice born from *Ocn^+/–^* mothers, *Cyp11b1* and *Cyp11b2* expression was normal, and steroidogenesis proceeded normally in the offspring.

This study, mostly performed in the mouse, has potential relevance to human biology. Circulating osteocalcin levels are higher in men than in women, whereas circulating cortisol levels are similar in both sexes (30, 31). This discrepancy does not affect our conclusions, since the pool of osteocalcin that is important to assure proper adrenal steroidogenesis is the embryonic pool. In fact, 2 lines of evidence suggest that osteocalcin regulation of adrenal growth and steroidogenesis may exist in humans. First, a recent GWAS showed that an increase in systolic or diastolic blood pressure correlated with an increase in osteocalcin levels (32). Second, and more directly, this same study found that osteocalcin could enhance adrenal steroidogenesis in nonhuman primates. That we have been unable so far to identify individuals harboring a loss-of-function mutation in GPR158 through the screening of multiple databases is consistent with the notion that GPR158 seems intolerant to homozygous loss-of-function mutations (33). On the other hand, the fact that osteocalcin signaling in the adrenal gland regulates adrenal growth and steroidogenesis provides new molecular tools for an ongoing study aimed at elucidating the pathogenesis of the still poorly understood pathological bilateral adrenal cell proliferation (34).

The results presented in this study also have relevance for how steroidogenesis is regulated in mammals. Indeed, the regulation of testosterone and now corticosterone and aldosterone biosynthesis by osteocalcin, along with the modulation of 1α hydroxylation of 25 hydroxyl vitamin D_3_ by another bone-derived hormone, FGF23, identifies the skeleton as an organ that is necessary for the synthesis of 4 steroid hormones. Surprisingly, this is just as many steroidogenic pathways as are regulated by pituitary hormones, which further highlights the biological importance of bone as an endocrine organ.

The regulation of adrenal steroidogenesis by osteocalcin also illustrates the extent to which this hormone acts as a regulator of other regulatory molecules, whether they are peptide or steroid hormones, cytokines, or neurotransmitters (13, 20, 35). Together, the site of synthesis of osteocalcin and its wide array of regulatory functions have long begged the question of why all these functions are localized in bone? The need to coordinate the huge energy requirements of bone modeling and remodeling with food intake in conditions of food scarcity explains the coordinated regulation of bone turnover and energy metabolism (36). Moreover, osteocalcin is necessary to allow an acute stress response to proceed, promotes cognition, mobilizes glucose and fatty acids during exercise, and enhances exercise capacity (13, 20, 37). That all these functions are necessary to sense and/or escape danger suggests that osteocalcin may belong to an endocrine network orchestrating the response to danger in bony vertebrates. The role of adrenal steroid hormones in maintaining homeostasis and the regulation of their biosynthesis by osteocalcin are consistent with such a danger-sensing and fighting purpose.

## Methods

### Mice

Mc2r–/– (C57Bl/6J; ref. 24), Sf1-Cre (SV129; ref. 17), CamK2a-Cre (C57Bl/6J; JAX strain no. 005359), Gli1-CreERT (Swiss Webster; C57Bl/6J mixed; JAX strain no. 007913), Axin2-CreERT (C57Bl/6N; JAX strain no. 018867), Ocn–/– (SV129; ref. 38), Gpr158fl/fl (C57Bl/6J), and Gprc6a–/– mice (SV129; refs. 16, 27) have been described previously or were obtained from The Jackson Laboratory. For developmental deletion of Gpr158 in Gli1-CreERT or Axin2-CreERT, female floxed mice without Cre (Gpr158fl/fl; +/+) were time-mated with male mice carrying Cre (Gpr158fl/fl; +/Cre) and gavage-fed with 1 bolus of tamoxifen (4–5 mg) on E12.5, and mothers were sacrificed on E16.5 or E18.5 to collect embryos for further processing as described. For all experiments in Figures 1–8 and Supplemental Figures 1–8, only littermate control embryos or mice were used. WT mice of the indicated ages used for pharmacological experiments were obtained from The Jackson Laboratory or Taconic.

### Nonhuman primates

Adult female rhesus monkeys (*Macaca mulatta*) weighing 7–10 kg were individually housed in standard primate cages at the large animal facility National Institute of Immunology in New Delhi, India. Animals were maintained under standard environmental conditions (24°C ± 2.1°C, 55%–60% humidity, 12-hour light/12-hour dark photoperiod) and housed individually in stainless-steel nonhuman primate cages. Monkeys were injected at 1000 hours with recombinant human osteocalcin (Eli Lily; 13.5 ng/g body weight), and blood samples were collected by femoral venipuncture into Vacutainer blood collection tubes at different time points after injection. Blood was collected at room temperature, and serum was collected after centrifugation, divided into aliquots, and stored at –80°C until analysis. Thawed samples were not refrozen for further hormone analysis.

### Adrenal gland analysis

Adrenal glands were cleaned of excess fat, weighed, and frozen in liquid nitrogen or fixed in 4% paraformaldehyde for histological analysis. Adrenal weights were compared on the basis of the sum of both adrenal gland weights, normalized to body weight. We observed no significant difference in body weights between genotypes, and combined adrenal gland weights (left plus right adrenal glands) for each mouse are presented relative to their body weights in Figures 1–8 and Supplemental Figures 1–8.

### Hormone measurements in WT and mutant mice

Facial vein blood samples were obtained from mice at the indicated ages between 1745 hours and 1800 hours, within 10 seconds of handling of the mice to minimize stress-induced changes in adrenal steroid levels. Blood was collected in serum-separating tubes (Microvette 500 Z Gel, Starstedt), allowed to clot for 30 minutes, and centrifuged at 13,523*g* for 10 minutes at 4°C to obtain serum. Only 2 drops of blood were collected from each mouse, and the serum volume varied from 19–22 μL per mouse. Corticosterone and aldosterone levels were determined by ELISA (Abcam kits) in duplicate according to the manufacturer’s instructions. For adult mice, 2 μL serum was used for corticosterone and 5 μL for aldosterone measurements. Internal controls were used in each assay from a pooled serum sample collected from adult mice to calculate inter- and intra-assay variations. The interassay variation was less than 8 %–10%, and the intra-assay variation was less than 1%–2%.

### Measurement of hormone content in adrenal glands

#### Adult mice.

Fifty microliters lysate was used for corticosterone and aldosterone measurements. Internal controls were used in each assay from a pooled lysate collected from mice of the appropriate ages to calculate inter- and intra-assay variations. The interassay variation was less than 5%–10%, and the intra-assay variation was less than 1%–4%.

#### P1.

Pups were euthanized on ice between 1000 and 1200 hours. At least 3 pregnant females per genotype were used for analysis. The right adrenal glands were collected under a dissection microscope, snap-frozen in liquid nitrogen, and homogenized in 100 μL lysis buffer provided with the ELISA kits for 30 seconds using a hand-held pestle. In the same Eppendorf tube, an additional 200 μL lysis buffer was added for a total volume of 300 μL, followed by sonication for 30 seconds on ice. Samples were centrifuged at 4°C for 5 minutes at maximum speed, and supernatants were used for ELISAs to measure corticosterone and aldosterone levels using Abcam kits in duplicate according to the manufacturer’s instructions.

#### E18.5.

Embryos were euthanized on ice between 1000 hours and 1200 hours. At least 3 pregnant females per genotype were used for analysis. Both adrenal glands were collected under a dissection microscope, snap-frozen in liquid nitrogen, and homogenized in 75 μL lysis buffer provided with the ELISA kit for 20 seconds using a hand-held pestle to measure corticosterone levels. The lysate was placed in an Eppendorf tube followed by sonication for 10 seconds on ice. Samples were then centrifuged at 4°C for 5 minutes at maximum speed, and 5 μL supernatants were used for ELISAs to measure corticosterone and aldosterone levels using Abcam kits in duplicate according to the manufacturer’s instructions. Internal controls from a pooled lysate collected from embryos were used in each assay to calculate inter- and intra-assay variations. The interassay variation was less than 3%–8%, and the intra-assay variation was less than 1%–3%.

### Osteocalcin test in WT and mutant mice and hormone measurements

Animals were handled at least 5 days before the experiment. The animals were weighed at 1000 hours on the day of the experiment, tagged with markers, and randomized. Intraperitoneal injection of osteocalcin 30 ng/g or vehicle 0.1% BSA in PBS was performed at 1200 hours or 1800 hours. Two hours after injection, blood samples were collected from each animal (injections were done sequentially between 1200 hours and 1230 hours or 1800 hours and 1830 hours). Blood samples were obtained from the facial vein between 1400 hours and 1430 hours or 2000 hours and 2030 hours, within 10–15 seconds of handling the mice to minimize stress-induced changes in adrenal steroid levels. Different cohorts of animals were used for vehicle and osteocalcin injections, and samples were collected 1, 2, 6, and 24 hours after injection analysis. Blood was collected in serum-separating tubes (Microvette 500 Z Gel, Starstedt), allowed to clot for 30 minutes, and centrifuged at 13,523*g* for 10 minutes at 4°C to obtain serum. Only 2 drops of blood were collected from each mouse, and the serum volume varied from 19–22 μL per mouse. Circulating corticosterone and aldosterone levels were determined by ELISAs (Abcam) in duplicate according to the manufacturer’s instructions. For adult mice, 2 μL serum was used for corticosterone and 5 μL for aldosterone measurements. Internal controls from a pooled serum sample collected from adult mice were used in each assay to calculate inter- and intra-assay variations. The interassay variation was less than 8%–10%, and the intra-assay variation was less than 1%–2%.

### Blood pressure measurements

Mice of the genotypes indicated in the figures were weaned at 3 weeks of age and subjected to blood pressure measurements via a noninvasive tail-cuff plethysmography method (CODA 6 Non-invasive Mouse Blood Pressure Monitor; Kent Scientific) as previously described (39). Animals were acclimated to the measurement process over a 3-days training period (15 minutes per day). For a given session, the animals were placed in Kent Plexiglass holders and kept on the Kent heating stage at the L5 temperature setting, with blood pressure measurement cuffs placed on the base of the tail. After an additional 15-minute acclimation period on the heating stage, 15 consecutive blood pressure measurements were taken over the course of 30 minutes on the day of the experiment. The final 5 blood pressure measurements that were considered valid (e.g., no mouse movement, sufficient volume) were averaged to calculate systolic and diastolic blood pressure. All measurements were conducted between 0800 hours and 1700 hours.

### Measurement of K^+^ in plasma collected from facial vein

The Enterprise Point-of-Care (EPOC) (Element POC, Heska) was used to measure blood K^+^ concentrations in WT and mutant mice. Samples were collected from the facial vein in green top lithium-heparin tubes and mixed immediately to inhibit clotting. Any specimen with clotted blood was excluded from analysis. All analytes were measured immediately after specimen collection. The following biochemical variables were measured: pH, sodium (Na^+^), K^+^, ionized calcium (iCa2^+^), chloride ion (Cl^−^), and partial pressure of carbon dioxide (PCO_2_). Reference values (RVs) were established, and CIs were calculated around the upper and lower reference limits following the guidelines of the American Society for Veterinary Clinical Pathology (ASVCP). Outliers in the data were identified using Dixon’s and Tukey’s methods.

### Stress

All animals of the same batch were born within a 2-week interval and kept in mixed genotype groups of 2–5 females in the same cage under standard laboratory conditions (12-hour light/12-hour dark cycle, constant room temperature and humidity, and standard lab chow and water ad libitum). For each test, the mice were transported a short distance from the holding mouse facility to the testing room in their home cages. Mouse weights were between 22 g and 32 g. Unless otherwise indicated, a baseline blood sample was taken 48 hours prior to the onset of stress. In the case of the time course experiment, each time point represents a separate batch of mice exposed to the indicated period of stress. Stress was delivered by an experimentalist blinded to the genotypes or treatment of the mice under study. For TMT stress, food and water were removed immediately prior to TMT exposure. A cotton swab containing 10 μL TMT (300000368 , Scotts Canada Ltd.) was placed in the home cage for 15 minutes. Serum was collected after 15 minutes of TMT exposure, and the mice were transferred to a fresh home cage free of TMT odor.

### Gene expression analysis

For gene expression analysis, total RNA was isolated from whole adrenal glands of animals of each genotype using a QIAGEN RNA Isolation Kit, followed by quantification using NanoDrop (Thermo Fisher Scientific), and reverse transcription was performed with 1 μg RNA in a 20 μL volume. cDNA (1 μL) was used for quantitative real-time PCR (qRT-PCR) analysis of the respective genes using the SYBR Green method (Applied Biosystems), and 18s rRNA was used as an internal control. cDNA for the internal control was diluted 50–500 times to reach a Ct value within 5–6 cycles of the gene whose expression was being tested. *Akr1c18* was measured separately in virgin female and male mice before P26 and only in virgin female mice at older ages. qRT-PCR end-products were run on 2% agarose gels to confirm the specificity of the primers. Gene expression was reanalyzed via standard qRT-PCR in the linear range of amplification and run on a 2% agarose gel to confirm the change observed through the qRT-PCR.

The qRT-PCR primer sequences used for SYBR Green–based qRT-PCR assays were as follows: *Cyp11b1* forward: CAGATTGTGTTTGTGACGTTGC; *Cyp11b1* reverse: CGGTTGAAGTACCATTCTGGC; *Cyp11b2* forward: TGGCTGAAGATGATACAGATCCT; *Cyp11b2* reverse: CACTGTGCCTGAAAATGGGC; *Mc2r* forward: ACACCGCAAGAAATAACTCCG; *Mc2r* reverse: AGGAGGACAATCAAGTTCTCCA; *Agtr1a* forward: AACAGCTTGGTGGTGATCGTC; *Agtr1a*R:CATAGCGGTATAGACAGCCCA; *Agtr1b* forward: TGGCTTGGCTAGTTTGCCG; *Agtr1b* reverse: ACCCAGTCCAATGGGGAGT; *Agtr2* forward: AACTGGCACCAATGAGTCCG; *Agtr2* reverse: CCAAAAGGAGTAAGTCAGCCAAG; *Nr5a1* forward: TGCAGAATGGCCGACCAG; *Nr5a1* reverse: TACTGGACCTGGCGGTAGAT; *Ccna1* forward: TGATGCTTGTCAAATGCTCAGC; *Ccna1* reverse: AGGTCCTCCTGTACTGCTCAT; *Ccna2* forward: GCCTTCACCATTCATGTGGAT; *Ccna2* reverse: TTGCTGCGGGTAAAGAGACAG; *Ccnb1* forward: AAGGTGCCTGTGTGTGAACC; *Ccnb1* reverse: GTCAGCCCCATCATCTGCG; *Ccnb2* forward: GCCAAGAGCCATGTGACTATC; *Ccnb2* reverse: CAGAGCTGGTACTTTGGTGTTC; *Ccnd1* forward: GCGTACCCTGACACCAATCTC; *Ccnd1* reverse: CTCCTCTTCGCACTTCTGCTC*; Ccnd2* forward: GAGTGGGAACTGGTAGTGTTG; *Ccnd2* reverse: CGCACAGAGCGATGAAGGT; *Ccne1* forward: GTGGCTCCGACCTTTCAGTC; *Ccne1* reverse: CACAGTCTTGTCAATCTTGGCA; *Ccne2* forward: ATGTCAAGACGCAGCCGTTTA; *Ccne2* reverse: GCTGATTCCTCCAGACAGTACA; *Ccnf* forward: GTAGGTGTGCCAAGTGTTTCT; *Ccnf* reverse: TTCTGGGAGACTCAAGATGGTT; *Gpr158* forward: AGTGATGGCTGGTTTTCAGG; *Gpr158* reverse: TTGCAAATGCACTGATAGGC; *Gprc6a* forward: CCCAGTCTTGTCATACCCCAG; *Gprc6a* reverse: TGCTGTGTATCATAGCCAGAGT; *Crh* forward: CCTCAGCCGGTTCTGATCC; *Crh* reverse: GCGGAAAAAGTTAGCCGCAG; *Pomc* forward: CCTTGGTTTGGGTGGAAGATG; *Pomc* forward: GCGTTCTTGATGATGGCGTTC; and *18s* forward: CGCGGTTCTATTTTGTTGGT; *18s* reverse: AGTCGGCATCGTTTATGGTC.

### Histological and histomorphometric analyses

At the indicated ages, adrenal glands, brains, hypothalami, kidneys, and lungs were fixed in 4% paraformaldehyde (PFA) in PBS for 12 hours at 4°C, followed by dehydration and paraffin embedment. Histological analysis was performed as described previously (23). IHC for p-CREB was performed on 7 μm thick adrenal sections using previously described methods (16).

### ISH analysis

At the indicated ages, adrenal glands, brains, hypothalami, kidneys, and lungs from mice were fixed in 4% PFA in PBS for 12 hours at 4°C, followed be dehydration and paraffin embedment. For embryos, pregnant mothers were sacrificed at 1000 hours, and adrenal glands with kidney were exposed by removing the viscera and fixed for 12 hours, followed by dehydration and paraffin embedment. Five sections (7 μm thick) were used for ISH analysis performed with ACD RNAscope kits (RNAscope Multiplex Fluorescent Reagent Kit V2, Advanced Cell Diagnostics). All probes were obtained from the ACD RNAscope catalog. All images were taken using identical laser settings, and pseudocolor coding of the images was done for better comparative visualization.

### ACTH testing in WT and *Ocn^–/–^* offspring from *Ocn^+/+^*, *Ocn^+/–^*, and *Ocn^–/–^* mothers

Two-month-old WT and *Ocn^–/–^* mice born from *Ocn^+/+^*, *Ocn^+/–^*, or *Ocn^–/–^* mothers received vehicle (0.3% BSA in PBS) or recombinant ACTH (A0298, MilliporeSigma, 1 μg/g) via i.p., injection at 1000 hours. Injections were done sequentially between 1000 hours and 1025 hours. Blood samples were obtained between 1030 hours and 1100 hours from facial vein within 10 seconds of handling of the mice to minimize stress-induced changes in adrenal steroid levels. Blood was collected in serum-separating tubes (Microvette 500 Z Gel, Starstedt), allowed to clot for 30 minutes, and centrifuged at 13,523*g* for 10 minutes at 4°C to obtain serum. Only 2 drops of blood were collected from each mouse, and serum volumes varied between 10 μL and 20 μL per mouse. Circulating corticosterone and aldosterone levels were determined by ELISAs (Abcam) in duplicate according to the manufacturer’s instructions. Serum (2 μL) was used for corticosterone and 5 μL for aldosterone measurements with biological replicates. Internal controls from a pooled serum sample collected from adult mice were used in each assay to calculate inter- and intra-assay variations. The interassay variation was less than 7%–10%, and the intra-assay variation was less than 1%–2%.

### Osteocalcin injections in *Mc2r^+/–^* mothers during pregnancy

Recombinant mouse osteocalcin (300 ng/day) or vehicle was injected i.p. into pregnant mothers once per day at 1700 hours from E10.5 to E18.5 or from E10.5 to birth. When necessary, embryos were euthanized between 1100 hours and 1300 hours, unless otherwise indicated, for the collection of tail for genotyping. Adrenal glands were snap-frozen for hormone measurements or gene expression analysis or fixed in 4% PFA prepared in 0.1% diethylpyrocarbonate–treated (DEPC-treated) water for ISH analysis.

### Osteocalcin injections into *Ocn^–/–^* and WT mothers during pregnancy

Recombinant mouse osteocalcin (240 ng/day) or vehicle was injected i.p. into *Ocn^–/–^* or WT pregnant mothers once per day from E10.5 or E14.5 until birth. Offspring were euthanized at 2 months of age, and adrenal gland weights and hormone levels were analyzed for all endpoints.

### Osteocalcin ELISA

Mouse circulating osteocalcin levels were measured by ELISA as previously described (40). Plates were coated with either anti-GLA or anti-MID antibodies in Antibody Coating Buffer (ImmunoChemistry Technologies) for 12 hours at room temperature and then washed once (0.1% Tween-20 in 1× PBS) and coated with blocking buffer (3% BSA and 0.1% Tween-20 in 1× PBS) for 4 hours. Blocking buffer was decanted, and samples and standards were loaded and incubated at 4°C for 12 hours. The plates were then washed 5 times and incubated with an antibody directed against the C-terminus of osteocalcin conjugated to HRP and incubated for 1 hour. The plate was then washed 5 times (0.1% Tween-20 in 1× PBS) and developed in a 100 μL 1-Step Ultra TMB ELISA (catalog 34028, Pierce, Thermo Fisher Scientific), and the reaction was terminated with an equivalent volume of stop solution (1N HCl). In monkeys, circulating osteocalcin was measured using a human osteocalcin ELSA from BioLegend.

### ELISAs

Corticosterone, aldosterone, DHEAs, cortisol, and epinephrine ELISA kits were obtained from the Abcam, and assays were performed following the manufacturer’s instructions.

### Statistics

All values are shown as the mean ± SEM. Statistical parameters including the exact sample numbers, post hoc tests, and statistical significance are reported in every figure and the figure legends. Data were considered statistically significant when *P* < 0.05 using a 2-tailed, unpaired *t* test or 1-way ANOVA followed by Tukey’s post hoc test. Data were analyzed using GraphPad Prism 7 (GraphPad Software). For all panels in the figures, *P < 0.05 versus WT or control.

### Study approval

All mouse experiments were carried out in accordance with protocols approved by the Columbia University Animal Ethics Committee (protocol no. AABC4500). The monkey study was approved by the Institutional Animal Ethics Committee of the National Institute of Immunology (NII) (file no. 25/31/2017-CPCSEA/VKY). All experimental methods were performed in accordance with the Committee for the Purpose of Control and Supervision of Experiments on Animals India (CPCSEA) guidelines and regulations under the supervision of a professional veterinarian at the primate research facility of NII (New Delhi, India). All animal experiments and reporting adhere to Animal Research: Reporting of In Vivo Experiments (ARRIVE) guidelines.

## Author contributions

VKY and GK conceived the study in its entirety. VKY, JMB, PS, and PN performed experiments. VKY, JMB, PS, PN, and GK analyzed and interpreted the data. VKY and GK wrote the manuscript.

## Supplementary Material

Supplemental data

## Figures and Tables

**Table 1:**
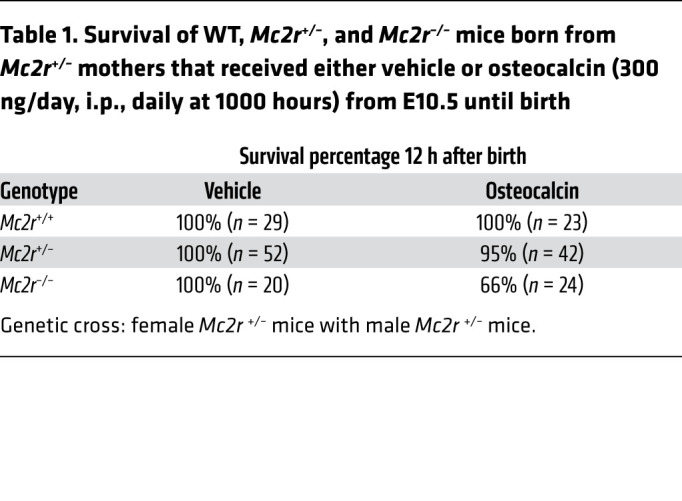
Survival of WT, *Mc2r^+/–^*, and *Mc2r^–/–^* mice born from *Mc2r^+/–^* mothers that received either vehicle or osteocalcin (300 ng/day, i.p., daily at 1000 hours) from E10.5 until birth

## Version 1 12/14/2021

In-Press Preview

## Version 2 02/15/2022

Electronic publication
