# Supplementary material for: Embryonic osteocalcin signaling determines lifelong adrenal steroidogenesis and homeostasis in the mouse
Source: J Clin Invest. 2022 Feb 15;132(4):e153752. doi: 10.1172/JCI153752 (PMC8843753; doi:10.1172/JCI153752)
Supplement: Supplemental data [file jci-132-153752-s047.pdf]

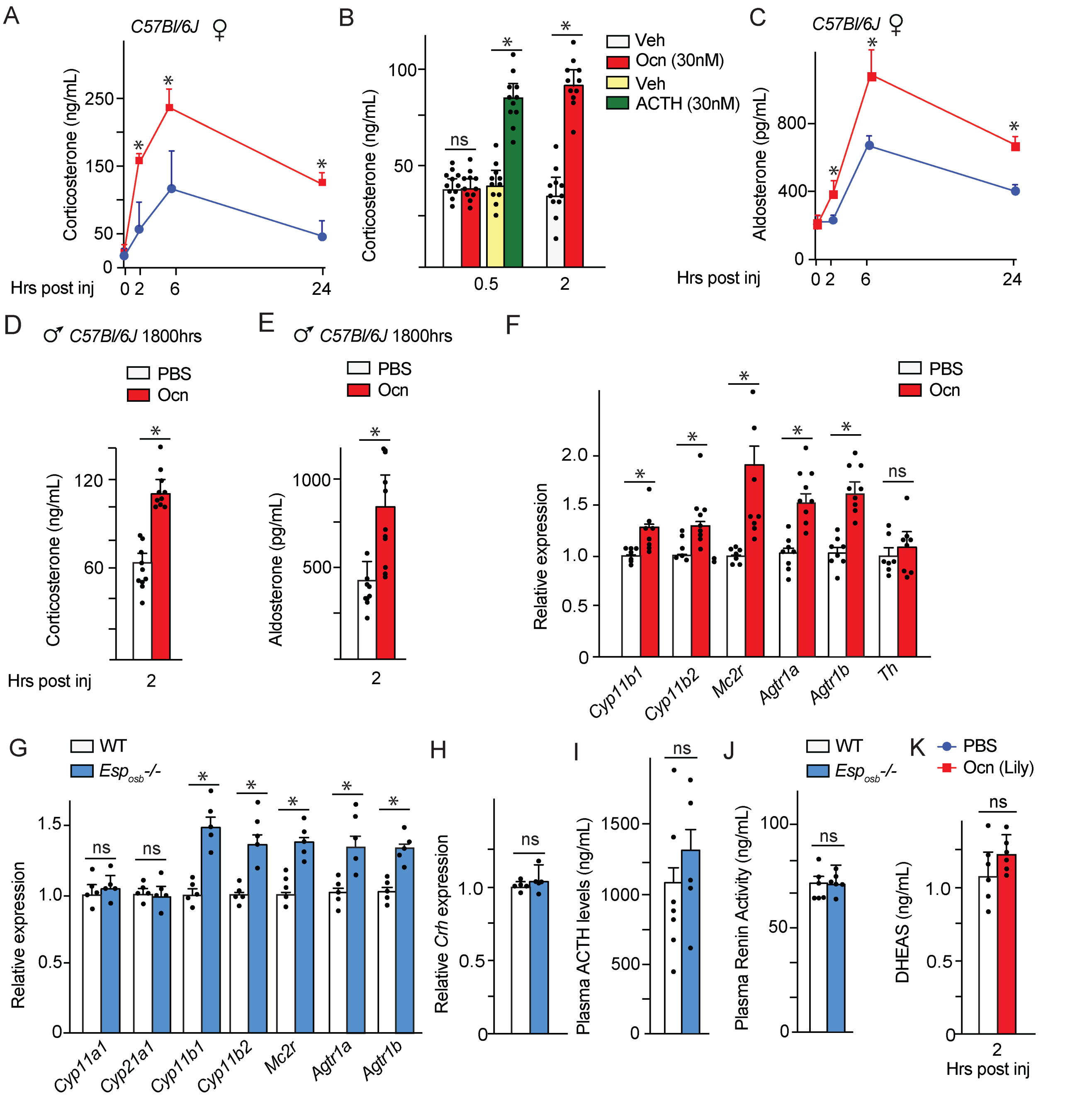

Legend to Figure S1 related to Figure 1. Osteocalcin increases circulating glucocorticoids and aldosterone in mice and monkeys. (A) Circulating corticosterone in 2 months-old WT *C57Bl/6J* female mice at different time points post osteocalcin injection. (B) Circulating corticosterone 0.5 and 2hrs post injection of osteocalcin (30nM) or ACTH (30nM) in WT 2 months-old mice. (C) circulating aldosterone in *C57Bl/6J* female WT mice at different time points post osteocalcin injection. (D-E) Circulating corticosterone (D) and aldosterone (E) in *C57Bl/6J* male mice 2hrs post osteocalcin injection at 1800hrs. (F) Steroidogenic gene expression in adrenal glands 2hrs post osteocalcin injection in WT mice. (G-J) Adrenal steroidogenic gene expression (G), *Crh* expression (H), plasma ACTH levels (I), plasma renin activity (J) in WT, Esp_{osb}-/- and Esp_{osb}-/-;*Ocn*+/-mice at 1800hrs. (K) Circulating DHEAS levels 2hrs after vehicle or human osteocalcin injection at 1000hrs in rhesus monkeys. * $p<0.05$. ns, not significant. Statistical analyses were conducted using 1-way ANOVA followed by Tukey's post hoc test (A, C) or 2-tailed unpaired t test (B, D-K). n=10 or more each group for mice except for panels F-H (n=4 or more in each group); n=5 or more for rhesus monkeys.

Figure S1

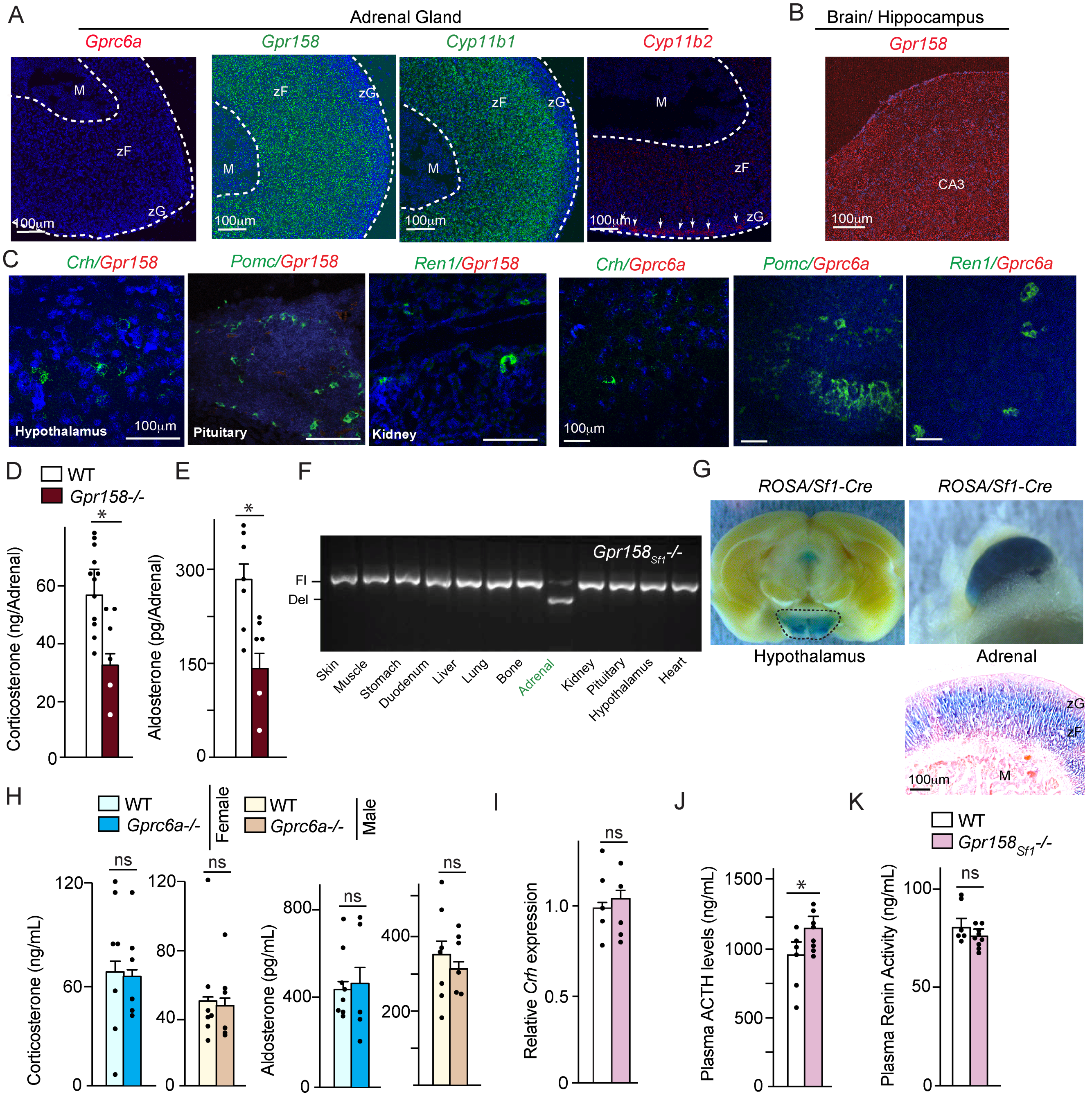

Legend to Figure S2 related to Figure 2. Osteocalcin signaling through Gpr158 in adrenal gland is necessary for adrenal steroidogenesis.(A) Lower magnification images (to be viewed with Figure 2C) of in situ hybridization analysis of *Gprc6a, Gpr158, Cyp11b1* and *Cyp11b2* expression in WT adrenal glands/hypothalamus/pituitary/ kidneys. (B) In situ hybridization analysis of *Gpr158* in the hippocampus (CA3 region). (C) In situ hybridization analysis of *Gpr158* (left panels) and *Gprc6a* (right panels) expression in WT hypothalamus, pituitary and kidneys. (D-E) Corticosterone (D) and aldosterone (E) intra-adrenal contents in 1 month-old WT and *Gpr158-/-* mice. (F) Recombination analysis on genomic DNA in different tissues collected from $Gpr158_{Sf1}$-/- mice. Floxed (Fl) and deletion (Del) bands are indicated. (G) Photomicrographs of Beta-galactosidase staining in the whole-mount brain and adrenal glands of *Sf1-Cre*+ mice crossed with ROSA reporter mice. (H) Circulating corticosterone and aldosterone in 2-month-old WT and *Gprc6a-/-* mice. (I-K) *Crh* expression in hypothalamus (I), plasma ACTH levels (J) and plasma renin activity (K) in WT and $Gpr158_{Sf1}$-/- mice. Statistical analyses were conducted using 2-tailed unpaired t test (D, E, H-K). n=6 more for each group. * p<0.05. ns, not significant.

Figure S2

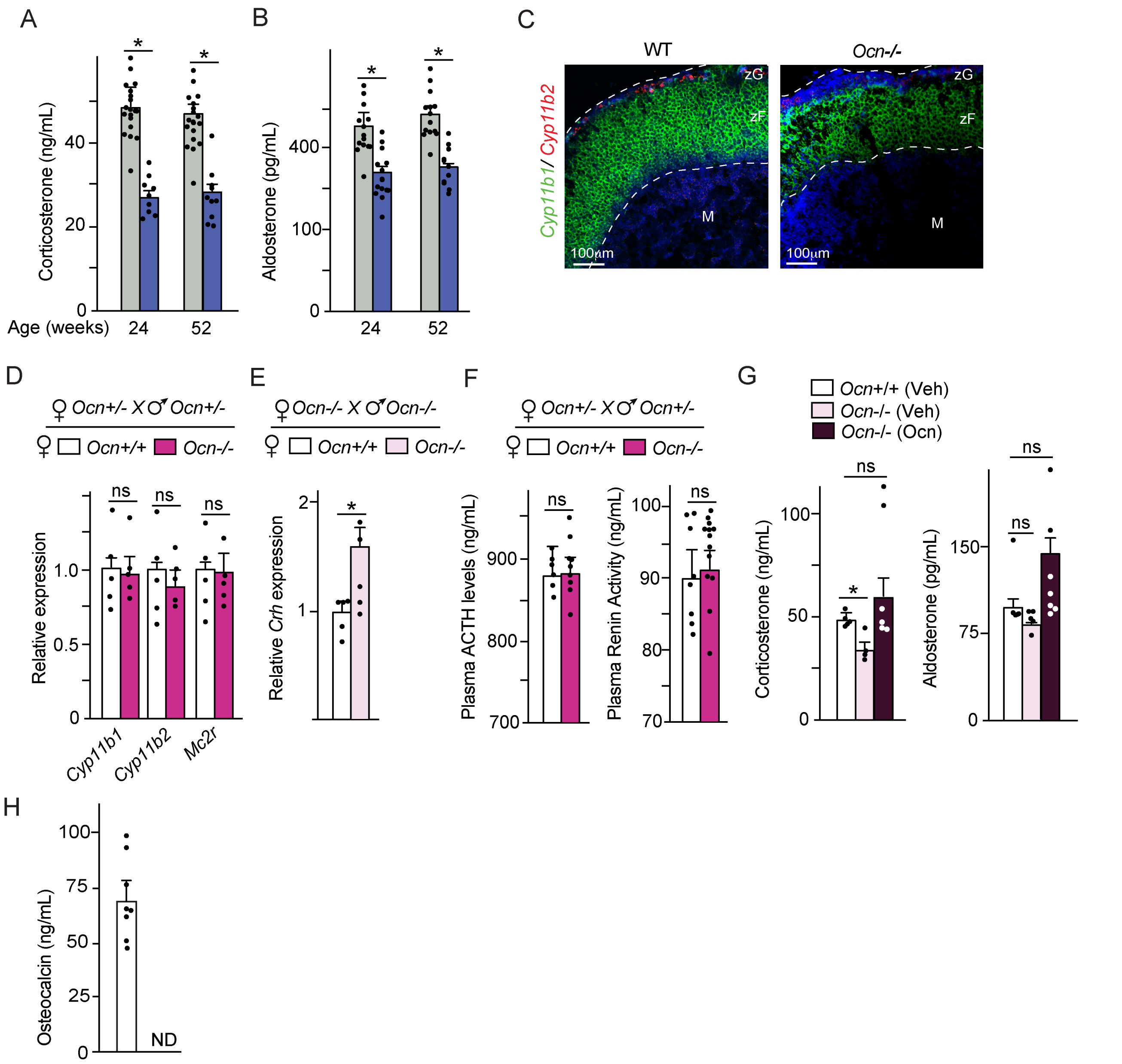

Legend to Figure S4 related to Figure 4. Embryonic osteocalcin promotes adrenal steroidogenesis and homeostasis in offspring. (A-B) Circulating corticosterone (A, 24 weeks- and 52 weeks-old) and aldosterone (B, 24 weeks- and 52 weeks-old) at 1800hrs in *Ocn*+/+ and -/- female and male mice born from *Ocn*+/+ or *Ocn*-/- isogenic parents. (C) *Cyp11b2* and *Cyp11b1* in situ expression analysis in adrenal glands in *Ocn*+/+ and -/- mice born from *Ocn*+/+ or *Ocn*-/- isogenic parents. (D) *Cyp11b1, Cyp11b2* and *Mc2r* adrenal expression in WT and *Ocn*-/- mice born from *Ocn*+/- parents. (E) *Crh* expression in hypothalamus of WT and *Ocn*-/- mice born from WT or *Ocn*-/- isogenic parents. (F) Plasma ACTH and renin activity levels in WT and *Ocn*-/- mice born from *Ocn*+/- parents. (G) Serum corticosterone and aldosterone levels in 2-months-old WT and *Ocn*-/- mice born from WT or *Ocn*-/- isogenic parents at 1000hrs following vehicle or recombinant Ocn (30ng/g BW) injection (i.p.,). (H) Circulating osteocalcin levels in 10 days-old WT and *Ocn*-/- mice born from *Ocn*+/- parents. Statistical analyses were conducted using 2-tailed unpaired t test (A, B, D-F, H) or 1-way ANOVA followed by Tukey's post hoc test (G). * p<0.05. ns, not significant. n=6 or more in each group.

Figure S4

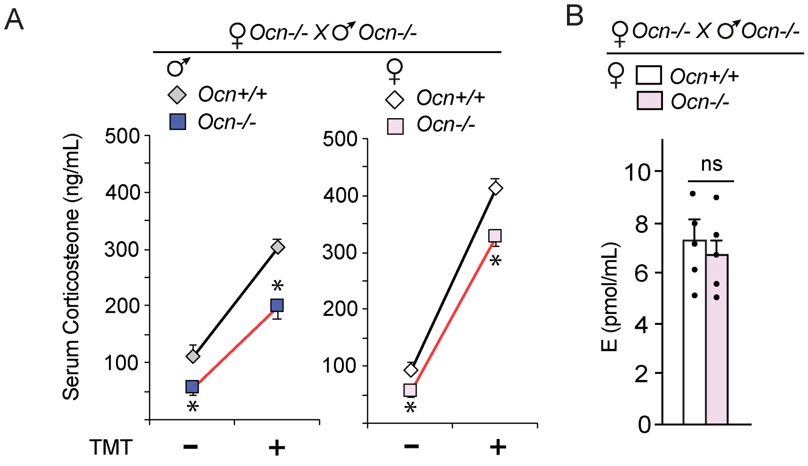

Legend to Figure S5 related to Figure 5. Embryonic osteocalcin promotes homeostasis in offspring. (A) Serum corticosterone levels at baseline and 30 minutes following exposure to a stressor (TMT) in male and female WT and *Ocn*-/- mice born from WT or *Ocn*-/- isogenic parents. (B) Serum epinephrine levels in 2 months-old WT and *Ocn*-/- mice born from WT or *Ocn*-/- isogenic parents. Statistical analyses were conducted using 2-tailed unpaired t test (all panels). * p<0.05. ns, not significant. n=5 or more in each group.

Figure S5

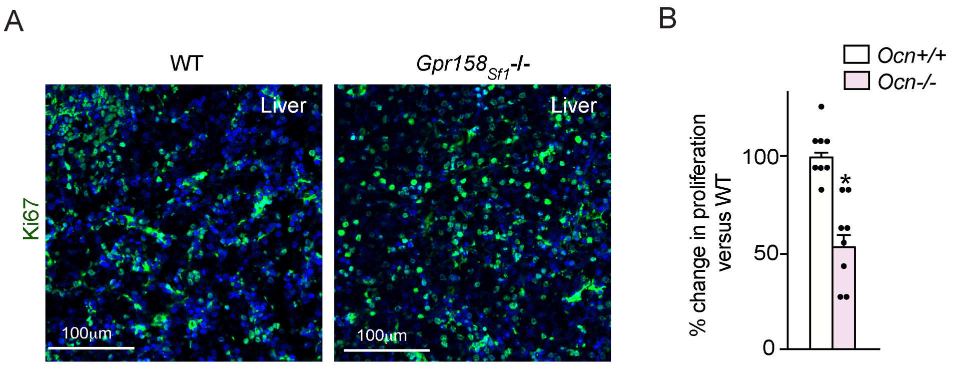

Legend to Figure S6 related to Figure 6. Embryonic osteocalcin signaling in adrenal glands promotes cell proliferation during development. (A) Proliferation analysis (Ki67 immunostaining) in liver sections of E18.5 WT and *Gpr158*$_{Sf1}$-/- embryos. (B) % change in proliferation in the adrenal glands of WT and *Ocn*-/- offspring born from *Ocn*-/- and WT isogenic parents. Statistical analyses were conducted using 2-tailed unpaired t test (B). *p<0.05. n=8 or more mice in each group.

Figure S6

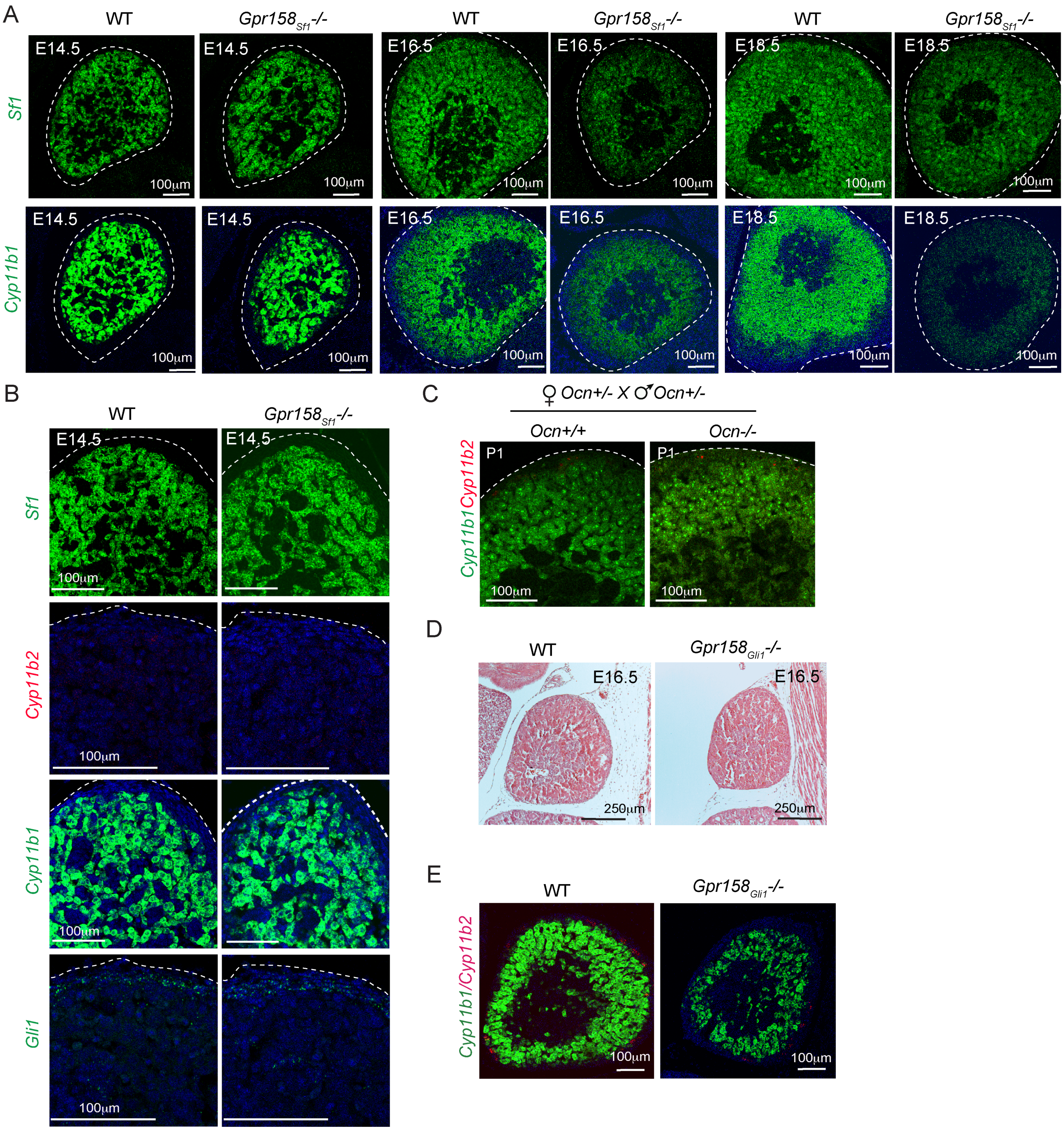

Legend to Figure S7 related to Figure 7. Embryonic osteocalcin signaling in adrenal glands is necessary to establish the steroidogenic program during development. (A) Lower magnification images (to be viewed with Figure 5B) of in situ hybridization analysis of *Sf1* and *Cyp11b1* expression in E14.5, E16.5 and E18.5 adrenal glands of WT and *Gpr158*$_{Sf1}$-/- embryos. (B) In situ hybridization analysis of *Sf1, Cyp11b2, Cyp11b1* and *Gli1* expression in E14.5 adrenal glands of WT and *Gpr158*$_{Sf1}$-/- embryos. (C) In situ hybridization analysis of *Cyp11b2* and *Cyp11b1* adrenal expression in P1 of WT and *Ocn*-/- pups born from *Ocn*+/- parents. (D) Hematoxylin and eosin-stained sections of adrenal glands of E16.5 WT and *Gpr158*$_{Gli1}$-/- embryos. (E) In situ hybridization analysis of *Cyp11b2* and *Cyp11b1* adrenal expression in E16.5 of WT and *Gpr158*$_{Gli1}$-/- embryos.

Figure S7

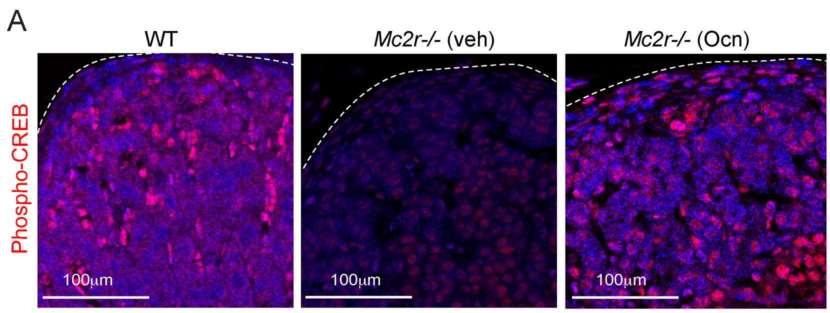

Legend to Figure S8 related to Figure 8. Osteocalcin induces adrenal steroidogenesis and growth in the absence of ACTH signaling. (A) Immunohistochemical localization of phospho-CREB in E18.5 WT and *Mc2r*-/- embryos collected from *Mc2r*+/- mothers that received either vehicle or osteocalcin (300ng/day) from E10.5 to 18.5.

Figure S8
